# Photoinduced electron transfer from rylenediimide radical anions and dianions to Re(bpy)(CO)_3_ using red and near-infrared light†
†Electronic supplementary information (ESI) available: Experimental details, detailed synthetic procedures, electrochemical characterization, fsTA, nsTA and fsIR data and fitting procedures; empirically calculated Gibbs free energy of electron transfer reactions; Arrhenius plot for **PDI^1–^-Phbpy-Re-Py**; and the DFT-calculated SOMO of Re(Phbpy˙^–^)(CO)_3_(PyPh). See DOI: 10.1039/c6sc05103k



**DOI:** 10.1039/c6sc05103k

**Published:** 2017-02-28

**Authors:** Nathan T. La Porte, Jose F. Martinez, Svante Hedström, Benjamin Rudshteyn, Brian T. Phelan, Catherine M. Mauck, Ryan M. Young, Victor S. Batista, Michael R. Wasielewski

**Affiliations:** a Department of Chemistry , Argonne-Northwestern Solar Energy Research (ANSER) Center , Northwestern University , Evanston , Illinois 60208-3113 , USA . Email: m-wasielewski@northwestern.edu; b Department of Chemistry , Argonne-Northwestern Solar Energy Research (ANSER) Center , Energy Sciences Institute , Yale University , New Haven , Connecticut 06520 , USA

## Abstract

A major goal of artificial photosynthesis research is photosensitizing highly reducing metal centers using as much as possible of the solar spectrum reaching Earth's surface. The radical anions and dianions of rylenediimide (RDI) dyes, which absorb at wavelengths as long as 950 nm, are powerful photoreductants with excited state oxidation potentials that rival or exceed those of organometallic chromophores. These dyes have been previously incorporated into all-organic donor–acceptor systems, but have not yet been shown to reduce organometallic centers. This study describes a set of dyads in which perylenediimide (**PDI**) or naphthalenediimide (**NDI**) chromophores are attached to Re(bpy)(CO)_3_ through either the bipyridine ligand or more directly to the Re center *via* a pyridine ligand. The chromophores are reduced with a mild reducing agent, after which excitation with long-wavelength red or near-infrared light leads to reduction of the Re complex. The kinetics of electron transfer from the photoexcited anions to the Re complex are monitored using transient visible/near-IR and mid-IR spectroscopy, complemented by theoretical spectroscopic assignments. The photo-driven charge shift from the reduced **PDI** or **NDI** to the complex occurs in picoseconds regardless of whether **PDI** or **NDI** is attached to the bipyridine or to the Re center, but back electron transfer is found to be three orders of magnitude slower with the chromophore attached to the Re center. These results will inform the design of future catalytic systems that incorporate RDI anions as chromophores.

## Abstract 2

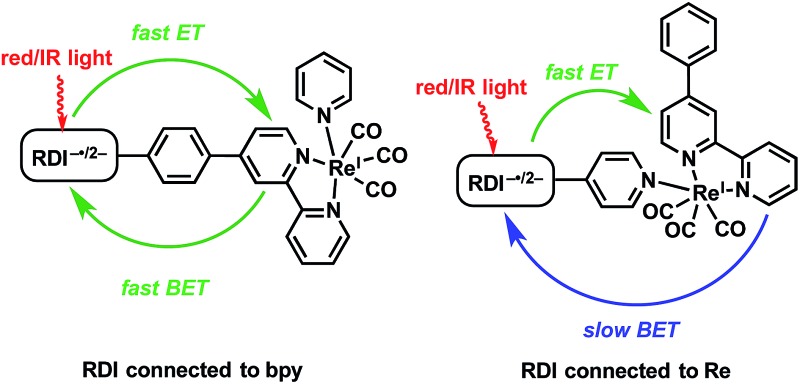
Photoinduced electron transfer dynamics are described for a set of dyads comprising rylenediimide anion chromophores and a Re(bpy)(CO)_3_ metal center.

## Introduction

One desirable component in the development of artificial photosynthetic systems is a chromophore having an excited state capable of reducing thermodynamically demanding organometallic catalysts, while exploiting the majority of the solar spectrum available at Earth's surface, including low energy photons in the near-infrared region. In the field of organometallic photochemistry, highly reducing chromophores have been developed, but these chromophores often incorporate non-earth-abundant metals such as Ir,^1,2^ Pt,^3^ or Re,^4^ of which none absorb light at wavelengths longer than 550 nm.^5–9^ There are very few reports of such highly reducing chromophores being incorporated into donor–acceptor assemblies, with only one report to date of an Ir-based photosensitizer and a CO_2_ reduction catalyst, in which an Ir^III^(1-phenylisoquinoline)_2_(bpy) complex is attached to a Re(bpy)(CO)_3_ catalyst, and catalysis is achieved upon illumination at 480 nm.^10^ There have also been a handful of reports of donor–acceptor assemblies run at wavelengths as long as 600 nm that incorporate less potently reducing chromophores such as zinc or magnesium porphryrins^11^ and Os^III^(bpy)_3_.^12^

In the field of fully organic photochemistry, most highly reducing chromophores absorb in the blue or UV regions, while those that absorb in the longer-wavelength visible region are typically not powerful enough reductants to reduce carbon dioxide reduction catalysts. However, the radical anions and dianions of the rylenediimide (RDI) dyes perylene-3,4:9,10-bis(dicarboximide) (**PDI**) and naphthalene-1,4:5,8-bis(dicarboximide) (**NDI**) are much stronger reductants than their neutral counterparts, while their absorption spectra extend significantly into the red or even near-infrared.^13^ When stored under inert atmosphere, these chromophores are stable in organic or even aqueous solution.^14^ A similar strategy, in which a reduced chromophore is used to sensitize a Re(bpy)(CO)_3_ center, has been reported very recently by Neumann and co-workers in a system incorporating a reduced polyoxometalate as a long-wavelength-absorbing chromophore.^15^ This method, in which a highly reducing chromophore is easily generated using chemical or electrochemical means and subsequently used to drive a highly endothermic electron transfer reaction, is applicable not only to the study of carbon-dioxide reduction, but could also be employed to facilitate other transformations such as photoelectrocatalytic N_2_ reduction or photoredox reactions involving electron-rich organic substrates that are not easily reduced using typical photoredox chromophores.

When used as electron acceptors, rylenediimides have been incorporated into numerous donor–acceptor assemblies using well-developed synthetic procedures. In a limited number of reports, assemblies have been developed to use the excited states of the **PDI** and **NDI** radical anions as electron donors to other organic molecules,^16–19^ but to date there have been no similar reports of electron transfer from the excited closed-shell dianions of either molecule, nor have there been any reports of electron transfer from any excited **PDI** or **NDI** anionic species to organometallic acceptors. In the present report, we describe donor–acceptor complexes comprised of the radical anions or dianions of RDI chromophores bound to Re(bpy)(CO)_3_ metal centers. These complexes result in a photoreduction of the Re(bpy)(CO)_3_ metal center using long-wave visible (>600 nm) or near-infrared (950 nm) light.

In prior complexes run by visible light, reduction of the Re(bpy)(CO)_3_ center proceeds *via* quenching of the excited state of the chromophore by a sacrificial donor, followed by thermal electron transfer from the reduced chromophore to Re(bpy)(CO)_3_.^12,20^ One complex has been reported in which reduction does proceed *via* excited state electron transfer, but the bpy ligand on the Re center has been modified to lower its reduction potential far below the threshold at which CO_2_ reduction could theoretically occur.^21^ We have recently reported on a set of compounds in which a reduced naphthalenediimide chromophore photoreduces a Re(bpy)(CO)_3_ center through an intermediate diphenylanthracene acceptor.^22^ That work explored the effect of changing the attachment geometry of the diphenylanthracene to the Re center; however the binding between the **NDI** radical anion chromophore and the diphenylanthracene acceptor was not varied, so there was no change in the excited-state electron transfer kinetics. Furthermore, in that study, we only examined the **NDI** radical anion chromophore, while the present report also describes electron transfer to the Re(bpy)(CO)_3_ center from the excited states of both the **PDI** radical anion and dianion.

In the complexes presented in this report, the RDI anion chromophore is bound to the Re(bpy)(CO)_3_ center either through the bpy ligand or directly to the Re center through a pyridine ligand. The forward- and back-electron transfer kinetics of the complexes are investigated using a combination of femto- and nanosecond transient absorption spectroscopies in the visible/NIR (fsTA, nsTA) and mid-IR (fsIR) to observe the transient photoproducts of both the chromophore and the metal center, as complemented by detailed spectroscopic assignments provided by vibronically resolved time-dependent DFT (TD-DFT) calculations. The difference between the two architectures is notable: while both types of complexes exhibit ultrafast charge shift kinetics, the complexes in which the RDI anion is attached to the bpy ligand *via* a phenyl spacer have back-electron-transfer lifetimes in the tens of picoseconds, while the complexes in which the RDI anion is attached directly to the pyridine ligand on the Re center have back-electron-transfer lifetimes in the nanosecond regime. In both cases, fsIR and DFT confirms that the reduction in the Re(bpy)(CO)_3_ complex is centered on the bpy ligand, known to be the first step in the photocatalytic mechanism of CO_2_ reduction by such complexes.

The RDI-pyridine-ligated chromophore will be of less utility than the RDI-bpy-ligated chromophore, since in order to initiate CO_2_ binding, the pyridine ligand must dissociate from the Re center, and this dissociation would prevent catalytic turnover. Nevertheless, our observation of the difference in electron transfer rate between complexes in which the chromophore is bound to the bipyridine ligand and complexes in which the chromophore is bound directly to the Re center is useful for the design of future catalytic systems for two reasons. First, Ishitani and co-workers have developed Re(diimine) complexes that are catalytic for CO_2_ reduction and which contain redox-innocent ligands such as phosphines that do not dissociate during catalysis, to which a chromophore could be attached.^23^ Second, Kubiak and co-workers recently reported a Mn-based CO_2_ reduction catalyst that contains only redox-innocent isocyanide ligands that do not dissociate during catalysis.^24^ These two reports suggest that attaching a reduced RDI chromophore to either the redox-active bpy ligand or directly the Re center holds promise, and consequently it is valuable to understand the effect that these different attachment motifs have on the electron transfer behavior of the complex.

## Experimental procedures

### Femtosecond transient absorption spectroscopy (fsTA)

Femtosecond transient absorption experiments were performed employing a regeneratively amplified Ti:sapphire laser system operating at 828 nm and a 1 kHz repetition rate as previously described.^25,26^ The output of the amplifier was frequency-doubled to 414 nm using a BBO crystal and that light was used to pump a laboratory-built collinear optical parametric (OPA) amplifier^27^ for visible-light excitation or a commercial non-collinear optical parametric amplifier (TOPAS-White, Light-Conversion, LLC) for NIR excitation. Approximately 1–3 mW of the fundamental was focused onto a sapphire disk to generate the visible white-light probe spanning 430–850 nm, or into a 5 mm quartz cuvette containing a 1 : 1 mixture of H_2_O : D_2_O to generate a UV/visible white light probe spanning 385–750 nm, or onto a proprietary medium (Ultrafast Systems, LLC) to generate the NIR white-light probe spanning 850–1620 nm. The total instrument response function was 300 fs.

Experiments were performed at a randomized pump polarization to suppress contributions from orientational dynamics. Spectral and kinetic data were collected with a CMOS or InGaAs array detector for visible and NIR detection, respectively, and an 8 ns pump-probe delay track (customized Helios, Ultrafast Systems, LLC). Transient spectra were averaged for at least 3 seconds. All spectra were acquired in DMF solution. Samples had an absorbance of 0.2–0.7 at the excitation wavelength and were irradiated in 2 mm quartz cuvettes with 0.4–0.8 μJ per pulse focused to a ∼0.2 mm diameter spot. Samples were stirred to avoid effects of local heating or sample degradation. The samples were prepared in the glovebox and degassed by multiple freeze–pump–thaw cycles prior to analysis.

### Nanosecond transient absorption spectroscopy (nsTA)

Nanosecond transient absorption experiments were performed using the femtosecond excitation beam described above and a commercial spectrometer (Eos, Ultrafast Systems, LLC) utilizing a photonic crystal fiber ultra-broadband probe source. The pump polarization was randomized to suppress rotational dynamics. Samples were stirred to avoid effects of local heating or sample degradation.

### Femtosecond transient mid-IR absorption spectroscopy (fsIR)

Femtosecond transient mid-IR absorption spectroscopy was performed using a commercial Ti:sapphire oscillator/amplifier (Solstice 3.5 W, Spectra-Physics) to pump two optical parametric amplifiers (TOPAS-C, light conversion), one which provided a 100 fs, 2 μJ excitation pulse at 705 or 605 nm and the other which provided 100 fs probe pulses from 2150–1800 cm^–1^. The overall instrument response was 0.3 ps. The spectra were acquired with a liquid N_2_-cooled dual channel (2 × 64) MCT array detector that is coupled to a Horiba iHR320 monochromator as part of a Helios-IR spectrometer (Ultrafast Systems, LLC) with a 300 l mm^–1^ grating (3 cm^–1^ resolution). Samples were prepared in DMF contained in a liquid demountable cell (Harrick Scientific) with CaF_2_ windows and a 500 μm or 630 μm Teflon spacer. The sample cell was mounted on a motorized stage and rastered during acquisition to reduce sample degradation.

### Synthesis

DCM and MeOH used for synthesis were obtained from Fisher Scientific and used as received. Acetonitrile, toluene, DMF and THF used for synthesis and spectroscopic experiments were dried on a commercial system (GlassContour, Laguna Beach, CA). For spectroscopy, DMF was further transferred under argon into a N_2_-filled glovebox (MBraun Unilab) for use and storage. Commercially available reagents were purchased from Sigma-Aldrich and used as received. Compounds were reduced in the glovebox using tetrakisdiaminoethylene (TDAE) from Tokyo Chemical Industries or CoCp_2_ from Sigma-Aldrich.

**Scheme 1:**
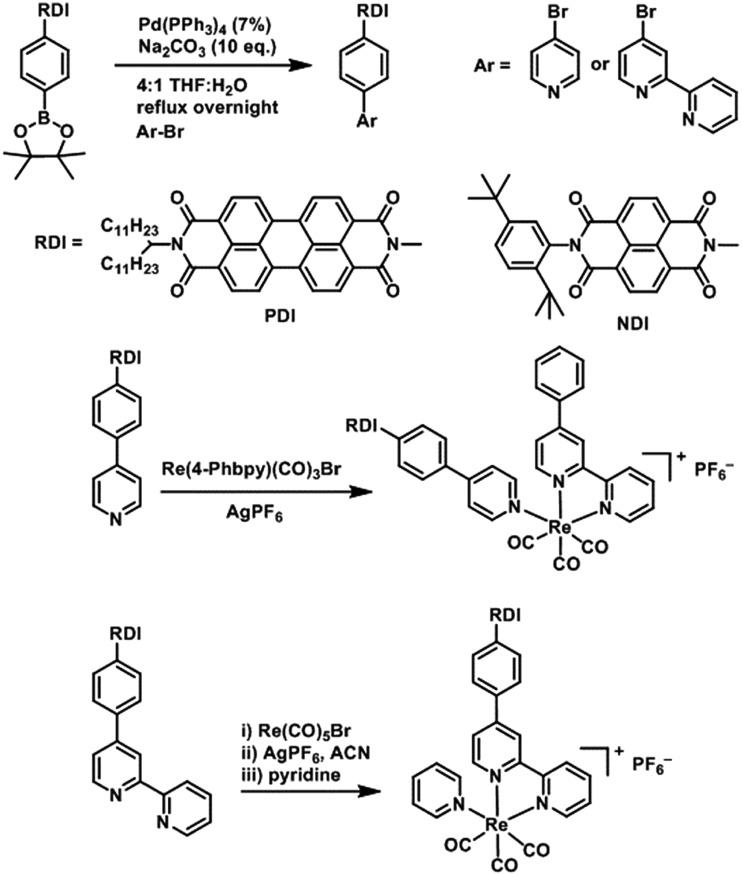
Synthesis of **RDI-Phbpy-Re-Py** and **Phbpy-Re-PyPhRDI**.

**PDI** and **NDI** chromophores were linked to Re(bpy)(CO)_3_ fragments both through the bipyridine ligand and through the pyridine ligand. Synthesis of the RDI-containing Re complexes was accomplished through Suzuki coupling of the appropriate **RDI-Ph-Bpin** to 4-bromopyridine or 4-bromobipyridine, followed by reaction with the appropriate Re precursor (Scheme 1) and purification on silica to produce the desired complex. Detailed synthetic procedures and characterization of compounds are provided in the ESI.†

## Results

**Fig. 1:**
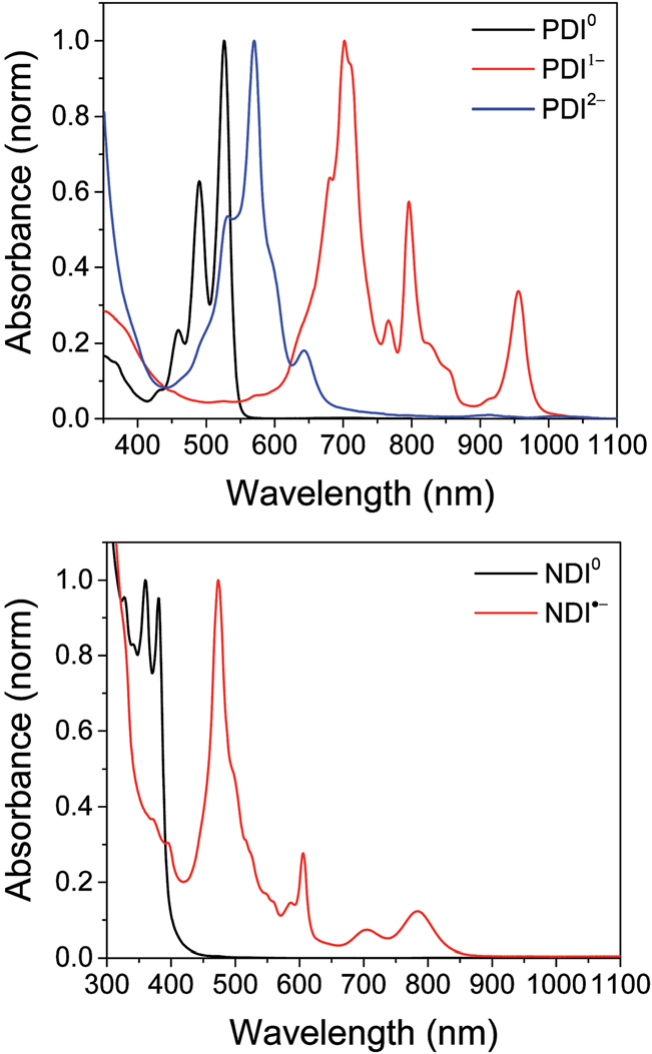
Top: Spectra of **PDI-Phbpy-Re-Py** in the neutral (black), PDI^–^˙ (red) and PDI^2–^ (blue) states. Bottom: Spectra of **Phbpy-Re-PyPhNDI** in the neutral (black) and NDI^–^˙ (red) states.

The electrochemical redox potentials of the complexes and model complex Re(Ph-bpy)(CO)_3_(py) are shown in Table 1. Attachment of the RDI chromophore has a very minor effect on the bpy-centered and Re-centered reductions of the complexes, indicating that there is little electronic communication between the RDI and Re(bpy) moieties. Titration with the appropriate reductant under an inert atmosphere reduced the RDI moiety to RDI^*n*–^. TDAE (*E* = –0.60 V *vs.* SCE)^28^ allowed access to the RDI^–^˙ states without the risk of formation of RDI^2–^, while the use of CoCp_2_ (*E* = –0.87 V *vs.* SCE in CH_2_Cl_2_)^29^ allowed access to the PDI^2–^ states without the risk of reduction of the bpy ligand. It was not possible to obtain quantitatively reduced NDI^2–^ states of the complexes that incorporated that chromophore, because of the similar redox potentials of NDI^–^˙^/2–^ and bpy^0/–^˙ (–0.99 *vs.* –1.08 V, see Table 1). Because there are no wavelengths at which NDI^2–^ can be excited independently of background NDI^–^˙, experiments were not performed on the NDI^2–^ forms. Spectra of the RDI, RDI^–^˙ and RDI^2–^ states of the complexes are shown in Fig. 1, and their similarity to the reported spectra of uncomplexed neutral and reduced RDI^13^ similarly indicates weak electronic coupling between the two moieties.

**Table 1:** Electrochemical redox potentials for the complexes under study, as well as model compounds^*a*^

	*E*(RDI^0/–^)^*b*^ (V)	*E*(RDI^–/2–^)^*b*^ (V)	*E*(bpy^0/–^) (V)	*E*(Re^I/0^) (V)
**PDI** ^*c*^	–0.43	–0.70	—	—
**NDI** ^*c*^	–0.48	–0.99	—	—
**Phbpy-Re-Py**	—	—	–1.08	–1.53^*d*^
**PDI-Phbpy-Re-Py**	–0.51	–0.78	–1.11	–1.45^*d*^
**NDI-Phbpy-Re-Py**	–0.50	–1.01	–1.11	–1.47^*d*^
**Phbpy-Re-PyPhPDI**	–0.47	–0.74	–1.06	–1.47^*d*^
**Phbpy-Re-PyPhNDI**	–0.50	–1.05	–1.10	–1.47^*d*^

^*a*^All electrochemical experiments were performed in DMF with 0.1 M TBAPF_6_, using a platinum disk working electrode, a platinum wire counter electrode, and a silver wire pseudoreference electrode. Potentials were referenced to a ferrocene internal standard (*E*(Fc^+/0^) = 0.45 V *vs.* SCE) and are given *versus* the saturated calomel electrode (SCE).

^*b*^RDI^–^ is equivalent to the RDI^–^˙ radical anion.

^*c*^From ref. 13.

^*d*^Irreversible, *E*_peak_ from CV given.

For femtosecond transient absorption (fsTA) experiments, the complexes could also be reduced in a spectroelectrochemical cell, resulting in identical steady-state spectra, albeit with a large scattering background due to the presence of the Pt mesh working electrode. The fsTA spectra obtained from electrochemically reduced samples of **PDI-Phbpy-Re-Py** were found to be identical to those obtained from chemically reduced samples.

Shown in Fig. S1 in the ESI† are the visible and NIR transient absorption spectra of the uncomplexed RDI^*n*–^ chromophores. They display negative Δ*A* features corresponding to the loss of ground state absorptions and appearance of excited state stimulated emission, and positive Δ*A* features corresponding to excited state induced absorptions. For the doublet radical anions PDI^–^˙ and NDI^–^˙, these features all decay concurrently with lifetimes of 145 ± 15 ps and 141 ± 7 ps, respectively, consistent with earlier reports.^13^ For the singlet dianion PDI^2–^, the decay of the singlet excited state features is accompanied by the rise of new features which live for tens of nanoseconds and are presumed on the basis of their lifetime to correspond to the dianion triplet excited states. The lifetime of the PDI^2–^ singlet excited state has been reported as 6.5 ns.^14^

**Fig. 2:**
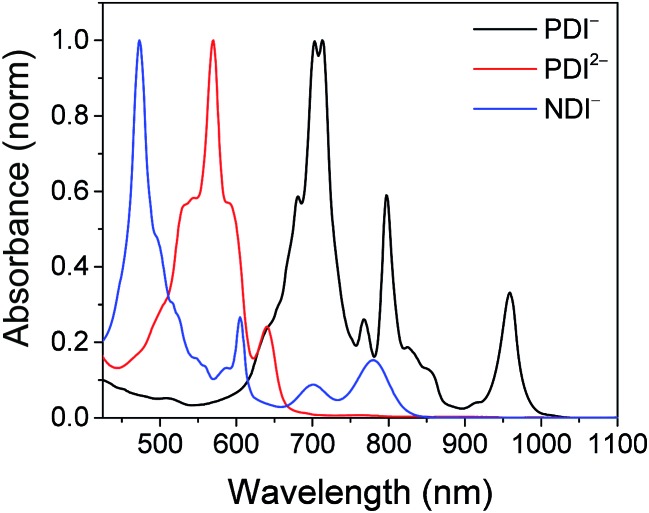
Spectra of chemically reduced samples of C23_2_-PDI (black: radical anion, reduced with TDAE, red: dianion, reduced with CoCp_2_) and 2,6-di^*t*^Bu-Ph-NDI-Ph (blue: radical anion, reduced with TDAE) in DMF.

Excitation of the RDI^*n*–^ moiety of each complex leads to instantaneous bleaching of the RDI^*n*–^ ground state absorption bands and formation the RDI^*n*–^ excited state bands. The lifetime of the excited state was diminished relative to the native RDI^*n*–^ chromophores, and at later times, bands characteristic of the RDI^(*n*–1)–^ states are observed. For example, Fig. 2A shows the time evolution of the transient absorption spectra for **Phbpy-Re-PyPhPDI^1–^**, showing induced absorptions of PDI^–^˙* at 459 nm and 550–630 nm, ground state bleaching of PDI^–^˙ centered at 700 nm and 797 nm, overlapped ground state bleach and stimulated emission bands at 959 nm and stimulated emission bands of PDI^–^˙ at 1100 nm and 1300 nm which decay to new induced absorptions of PDI^0^ at 490 nm and 525 nm. Similar spectra for the other molecules in the study are shown in Fig. S2–S14.† The lifetimes of the excited states of the various complexes are given in Table 2.

**Fig. 3:**
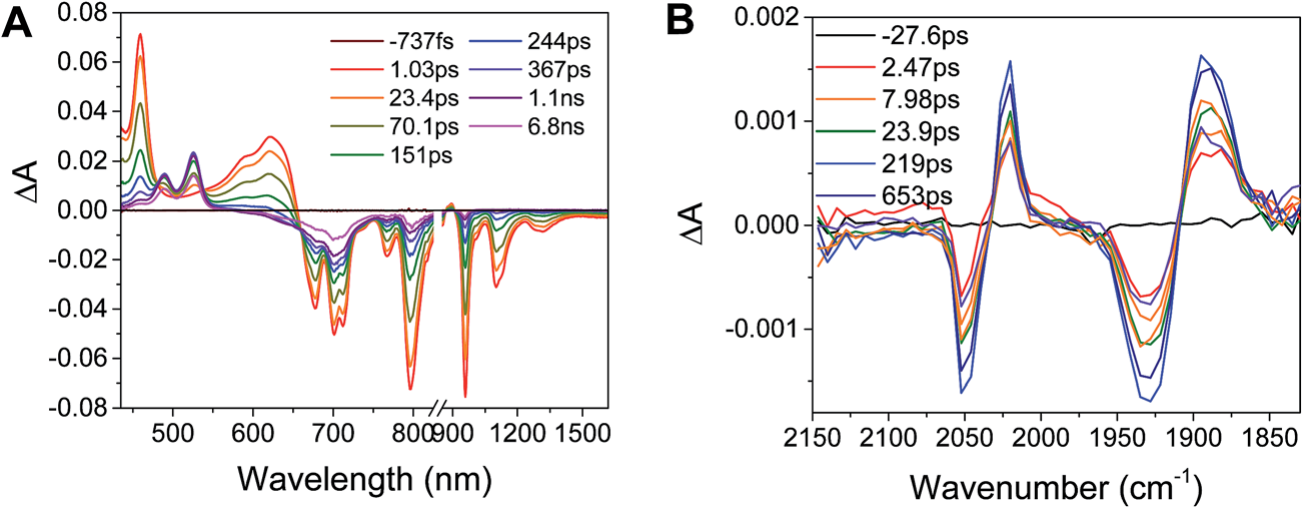
(A) Transient absorption spectrum of **Phbpy-Re-PyPhPDI^1–^** (*λ*_ex_ = 950 nm). (B) Time-resolved mid-IR spectrum of **Phbpy-Re-PyPhPDI^1–^** (*λ*_ex_ = 704 nm).

**Table 2:** Rates and Gibbs free energies of electron transfer reactions discussed in this study^*a*^

	RDI^*n*–^	*τ*_obs_ (ps)	*τ*_ET_ (ps)^*c*^	*k*_ET_ (s^–1^)	Δ*G* (eV)	*τ*_BET_	*k*_BET_ (s^–1^)	Δ*G*_BET_ (eV)
**PDI-bpy-Re-py**	1–	53.5 ± 1.4	23.4^*b*^	5.3 × 10^10^	–0.81	53.5 ± 1.4 ps	1.87 × 10^10^	–0.49
	2–	<0.3	<0.3	>3.33 × 10^12^	–1.47	107 ± 1 ps	9.35 × 10^9^	–0.25
**NDI-bpy-Re-py**	1–	0.4 ± 0.1	0.4	2.49 × 10^12^	–1.10	31.8 ± 0.8 ps	3.14 × 10^10^	–0.50
**Phbpy-Re-PyPhPDI**	1–	105 ± 0.9	381	2.6 × 10^9^	–0.82	17.1 ± 0.2 ns	5.85 × 10^7^	–0.48
	2–	0.9 ± 0.2	0.9	1.11 × 10^12^	–1.49	268 ± 1 ns	3.73 × 10^6^	–0.23
**Phbpy-Re-PyPhNDI**	1–	1.5 ± 0.1	1.5	6.6 × 10^11^	–1.11	29.7 ± 0.2 ns	3.37 × 10^7^	–0.49

^*a*^ *τ*_ex_: observed excited state lifetime; *τ*_ET_: empirically calculated excited state electron transfer rate; *k*_ET_: calculated excited state electron transfer rate; Δ*G*_q_: driving force for RDI^*n*–^* → bpy electron transfer; *τ*_BET_: back electron transfer lifetime; *k*_BET_: back electron transfer rate; Δ*G*_BET_: Gibbs free energy for bpy^–^˙ → RDI^(*n*–1)–^ back electron transfer.

^*b*^Calculated based on the growth of the charge-shifted state as determined by the kinetics at 525 nm (see Fig. 6).

^*c*^ *τ*_0_ values obtained from the literature. PDI^–^: 145 ps;^13^ PDI^2–^: 6.5 ns;^14^ NDI^–^: 141 ps.^13^

In the femtosecond time-resolved mid-IR transient absorption (fsIR) experiments, the CO-stretching region of the Re(bpy)(CO)_3_ moiety (1850–2100 cm^–1^) was monitored. All complexes exhibited the same spectral features, with only the kinetics differing from complex to complex. FsIR spectra for **Phbpy-Re-PyPhPDI^1–^** are shown in Fig. 3B, and similar spectra for the other molecules in the study are shown in Fig. S2–S14.† After excitation, bleaching of the ground-state absorptions at 2046 cm^–1^ and 1928 cm^–1^ was observed, and induced absorptions were observed at 2020 cm^–1^ and 1885 cm^–1^.

## Discussion

**Fig. 4:**
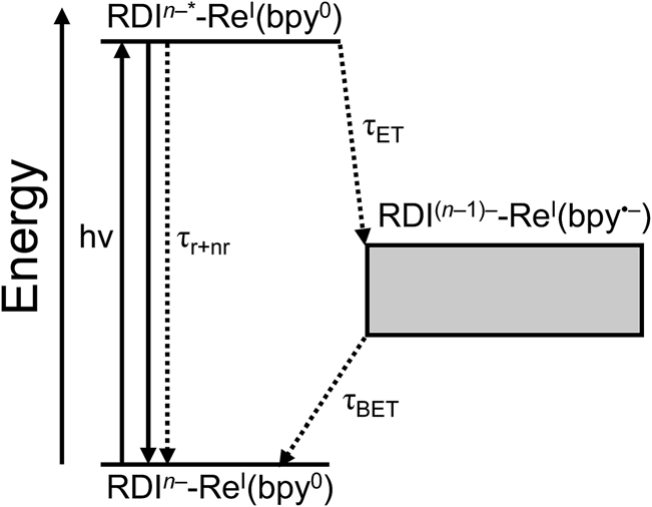
Jablonski diagram showing general scheme of electron transfer in RDI^*n*–^-Re(bpy) complexes after excitation. Energy of the charge-transfer state is shown as a range to reflect the different energies of different complexes.

For every complex except **PDI^1–^-Phbpy-Re-Py**, which will be discussed further below, the fsTA and fsIR data fit to a model in which the excited state decays to a charge-shifted state with the lifetime shown in Table 2, which then undergoes back-electron transfer to regenerate the ground state. The back-electron transfer lifetime is <100 ps for the RDI-bipyridine-ligated complexes, and >10 ns for the RDI-pyridine-ligated complexes. Plots showing the kinetic analysis of the visible/NIR and mid-IR spectra and kinetic fits for each complex are shown in Fig. S2–S14.† A Jablonski diagram showing the general electron-transfer behavior of the complexes is shown in Fig. 4.

Observation of the induced absorptions of RDI^(*n*–1)–^ and concomitant growth of induced absorptions in the mid-IR characteristic of the bpy-localized reduction of the Re(bpy)(CO)_3_ moiety (see below) indicates that the excited states decay *via* electron transfer to the Re(bpy)(CO)_3_ moiety, as shown in eqn (1):

1 RDI^*n*–^*-Re(bpy) → RDI^(*n*–1)–^-[Re(bpy)]^–^

The Gibbs free energies for the forward and back electron transfer steps were calculated (for details see ESI†) and are shown in Table 2.

The lifetimes and rates of forward electron-transfer quenching *τ*_ET_ and *k*_ET_ are shown in Table 2 and were calculated based on the observed excited state lifetime *τ*_obs_ and the intrinsic lifetime of the chromophore *τ*_0_ = *τ*_*r*+*nr*_:2
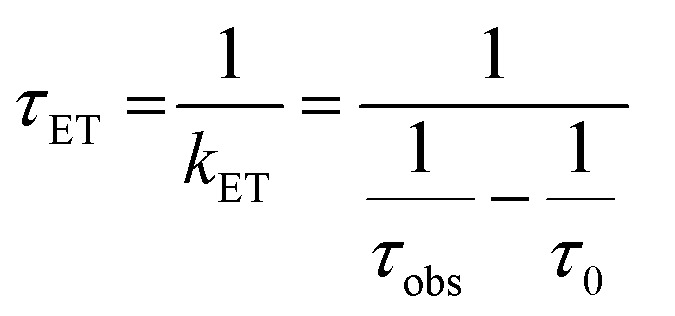



The time constants and rates of back-electron transfer *τ*_BET_ and *k*_BET_ can be calculated by observing the decay of the RDI^(*n*–1)–^ induced absorption bands, from the recovery of the RDI^*n*–^ ground state bleach, or from the decay of the induced absorptions and recovery of the ground state bleaches in the fsIR spectra. The data were fit as described in the ESI† (singular value decomposition global fitting or multiple-wavelength global fitting) and the back-electron transfer lifetime *τ*_BET_ extracted from the global fits. For every complex except **Phbpy-Re-PyPhPDI^2–^** and **Phbpy-Re-PyPhNDI^1–^**, the complexes exhibited monoexponential decay of the charge-shifted state features. The charge-shifted-state features in the complexes **Phbpy-Re-PyPhPDI^2–^** and **Phbpy-Re-PyPhNDI^1–^** exhibited biexponential decay kinetics, which will be discussed further below.

The time window of the fsIR data only extends to 7.5 ns, so it was not possible to obtain accurate fits for the fsIR data where lifetimes extended into the nanosecond regime. For the bpy-ligated complexes **RDI*^n^*^–^-Phbpy-Re-Py**, where lifetimes could be fit accurately, back-electron transfer lifetimes from fsIR matched those obtained from fsTA to within fitting error. For **Phbpy-Re-PyPhPDI^1–^**, the decay was also monoexponential but could not be fit accurately due to its long lifetime.

**Fig. 5:**
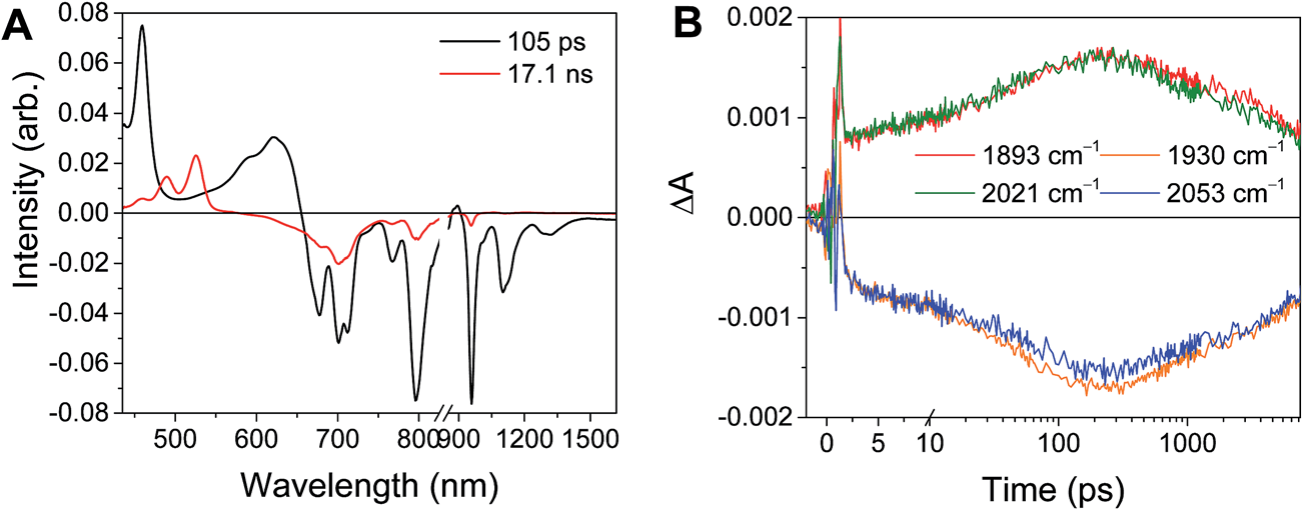
(A) Species-associated fit spectra of the fsTA data for **Phbpy-Re-PyPhPDI^1–^** shown in Fig. 2. Kinetic traces and fits are shown in Fig. S5.† (B) Kinetic traces of the fsIR data shown in Fig. 2.

For the complexes **Phbpy-Re-PyPhPDI^2–^** and **Phbpy-Re-PyPhNDI^1–^**, where the fsTA data exhibited biexponential decay kinetics, the fsIR data only exhibited a monoexponential decay which roughly corresponded to the longer component of the biexponential decay in the visible/NIR, but was too long to fit accurately. Successive fsIR scans of the same sample sometimes exhibited biexponential decay with a short component. We assign this short component to the product of photoreaction with trace oxygen under laser illumination, and posit that this product is also responsible for the short decay observed in the fsTA. Consequently, the lifetimes we report for back-electron transfer in the complexes **Phbpy-Re-PyPhPDI^2–^** and **Phbpy-Re-PyPhNDI^1–^** are the lifetimes of the long components of the visible/NIR TA. The back-electron-transfer lifetimes and rates are also shown in Table 2. A sample of the visible/NIR species-associated spectra and fsIR kinetics for **Phbpy-Re-PyPhPDI^1–^** in DMF are shown in Fig. 5A and B, respectively, and the full set of spectra and fits is included as Fig. S2–S14 in the ESI.†

### Electron transfer quenching of RDI^*n*–^*

Using fsIR, we can determine the nature of the charge-shifted state. Steady-state FTIR spectroscopy has been shown to allow facile differentiation among the various oxidation and ligand-field states of Re(bpy)(CO)_3_ complexes,^30–34^ and time-resolved mid-IR spectroscopy has been shown to be sensitive not only to the oxidation state of the Re center but also to transient electron density changes associated with excited states of a chromophoric ligand.^11,35–37^ A bathochromic shift of ∼25–45 cm^–1^ of the CO stretches corresponds exclusively to complexes in which the Re center remains Re^I^ while the bpy ligand is reduced to bpy^–^˙. A bpy-centered reduction is corroborated by DFT calculations of the reduced **[Re^I^(bpy)(CO)_3_]^–^**, see Fig. S17.† We therefore assign the charge-shifted state as RDI^(*n*–1)–^-Re^I^(bpy^–^˙)(CO)_3_.

Given that in the charge-shifted state of all the complexes, the electron is localized on the bipyridine ligand, the dramatic increase in back-electron-transfer lifetime upon switching from RDI-bipyridine to RDI-pyridine ligation can be rationalized. In the bipyridine-ligated complexes, the reduced bipyridine is separated from the oxidized chromophore by a single phenyl group. Indeed, DFT calculations of the **Phbpy-Re-Py** model compound show that upon reduction, the electron density extends onto the 4-phenyl group of 4-phenylbipyridine (see the LUMO in Fig. 7, and the anion SOMO in Fig. S17†). However, in the pyridine-ligated complexes, the through-space distance between the reduced bipyridine and the oxidized chromophore is quite significant (centroid-to-centroid distance of 16.6 Å in **Phbpy-Re-PyPhPDI** and 14.5 Å in **Phbpy-Re-PyPhNDI**), and the electron must either travel through intercalated solvent molecules in that space or through the difficult-to-reduce Re^I^ center in order to return. Similar lifetimes have been observed for systems in which back-electron transfer occurs over a comparable distance from a Re(bpy^–^˙)(CO)_3_ to an oxidized tryptophan linked through an pyridine-amido linker.^38^

**Fig. 6:**
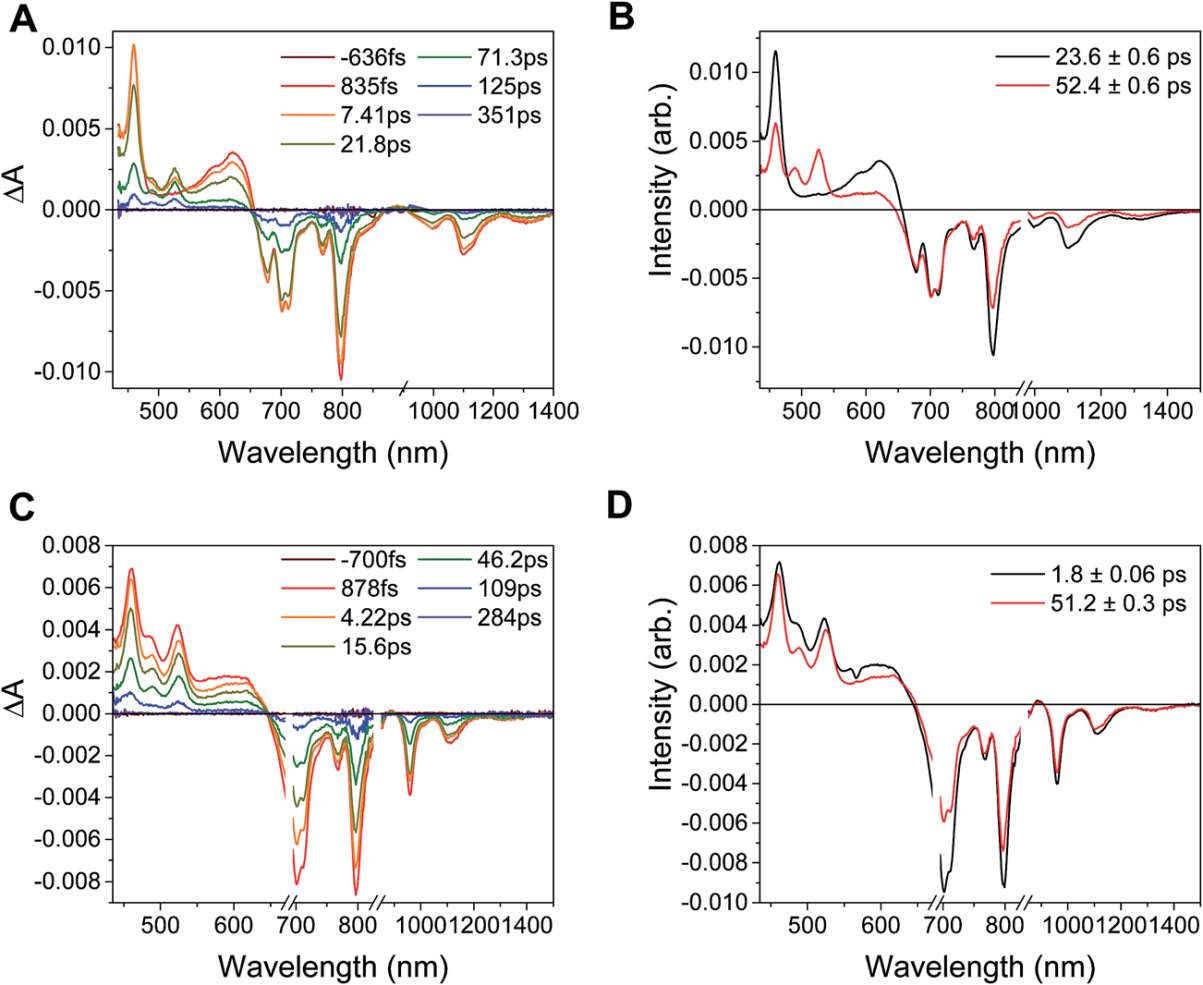
Top: (A) Transient absorption spectra and (B) decay-associated spectra for **PDI^1–^-Phbpy-Re-Py** (*λ*_ex_ = 950 nm). (C) Transient absorption spectra and (D) decay-associated spectra of those spectra for **PDI^1–^-Phbpy-Re-Py** (*λ*_ex_ = 680 nm).

In the complex **PDI^1–^-Phbpy-Re-Py**, the electron transfer kinetics are more complicated. When the complex is excited at 950 nm, the excited state features (Fig. 6B, black trace, consisting of absorbances at 459 nm and 550–650 nm and stimulated emission features at 1100 nm and 1300 nm) persist, but decrease in intensity while bands corresponding to the charge-shifted state grow in with a time constant of 23.6 ± 0.6 ps. These features (Fig. 6B, red trace) subsequently decay together with a lifetime of 52.4 ± 0.6 ps. When the complex is excited at 680 nm instead of 950 nm, bands characteristic of the charge-shifted state appear within the instrument response alongside excited-state features (Fig. 6D, black trace). This set of bands sharpens with a lifetime of 1.8 ps, producing a spectrum that is very similar to the final spectrum produced by 950 nm excitation (Fig. 6D, red trace), which then decays with a lifetime of 51.2 ± 0.3 ps.

**Fig. 7:**
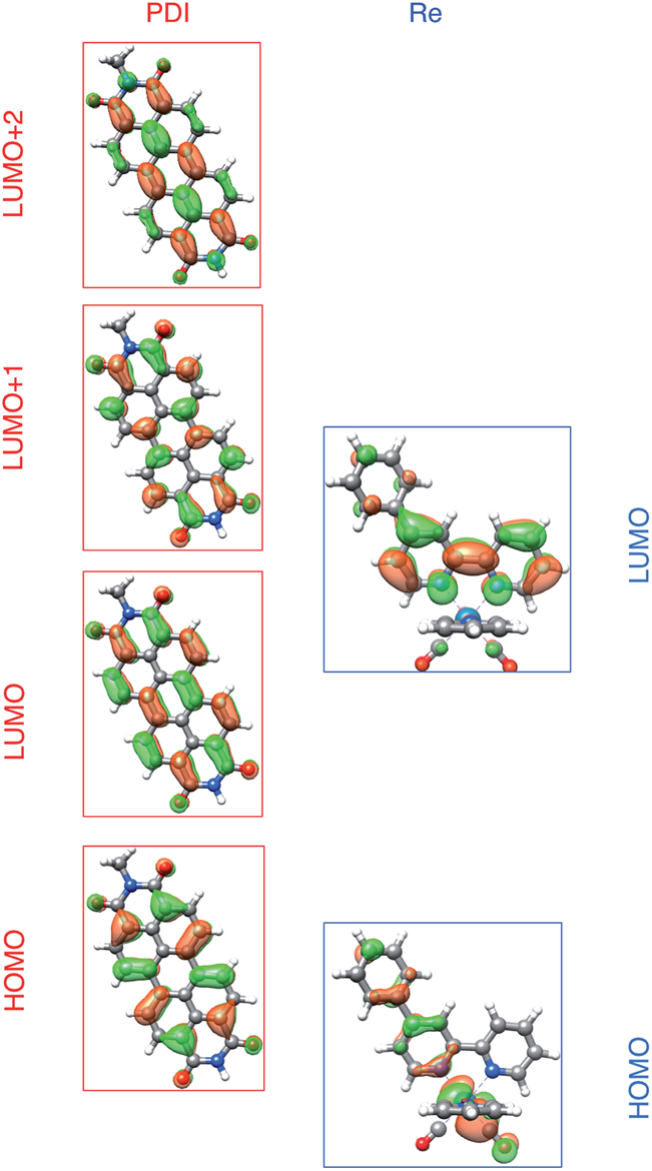
Frontier molecular orbitals of **PDI** (left) and [Re(Phbpy)(CO)_3_(py)]^+^ molecular fragments calculated using DFT (isovalue = 0.03). Frontier MOs of the full PDI–[Re(Phbpy)(CO)_3_(py)]^+^ dyad are shown in Fig. S15.†

To understand these results we must assign the absorption bands of PDI^1–^. Using TD-DFT calculations, these absorptions are shown to arise from three different electronic transitions of PDI^1–^, where the two different pump wavelengths promote distinct transitions. The relevant orbitals of the **PDI** chromophore fragment, calculated using DFT, are shown in Fig. 7. The orbitals of the full **PDI-Phbpy-Re-Py** dyad are shown in Fig. S15.† As reflected by the mostly unperturbed orbital energies given in Table S2,† these orbitals are essentially a combination of the MOs of the **PDI** and **Phbpy-Re-Py** fragments, with the exception of LUMO+4 orbital in which some mixing of the orbitals of each fragment occurs, see Fig. S15.†

**Fig. 8:**
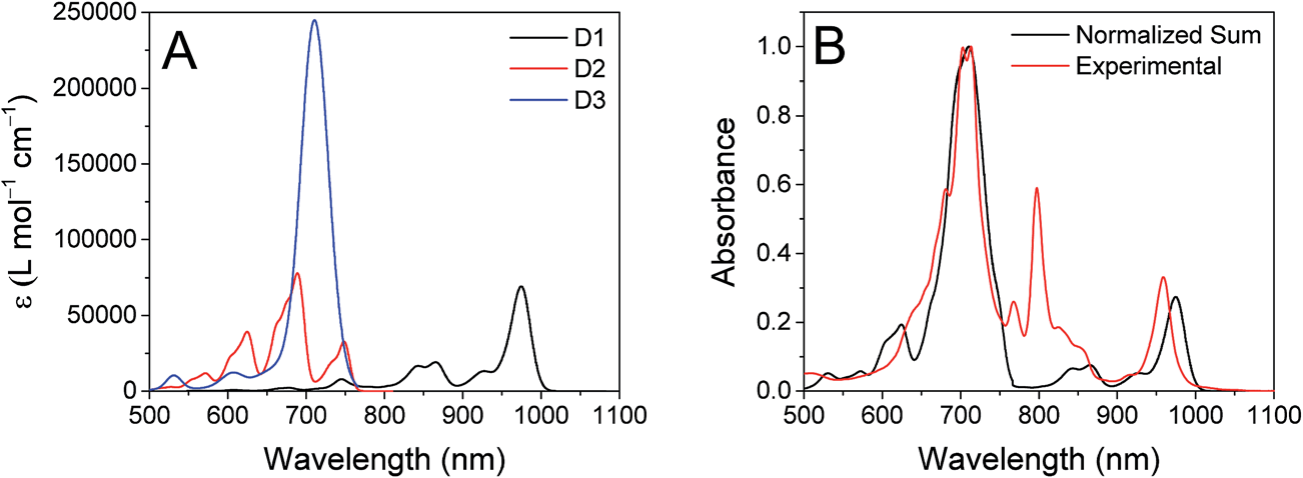
Left: Calculated vibronic transitions from the ground state to the D1 (black), D2 (red) and D3 (blue) states of PDI^–^. Right: Sum of the three calculated vibronic spectra (black) compared to the experimentally determined spectrum of PDI^–^, normalized to the maximum value.

The ten lowest-energy electronic transitions calculated by TD-DFT, are listed in Tables S3 and S4† for the Re(Phbpy)(py)(CO)_3_ and PDI^–^ fragments, respectively. The three lowest-energy transitions of PDI^–^ are labeled D1, D2, and D3 in order of increasing energy of their vertical, non-vibronic transitions, see Table 3. These excited states were structurally optimized, followed by excited-state numerical frequency calculations, permitting the calculation of vibronically resolved absorption spectra. The calculated vibronic spectra for each transition are shown in Fig. 8A, and the sum of the three transitions is compared to the experimentally determined spectrum of PDI^–^ in Fig. 8B, exhibiting excellent agreement of the ∼700 nm and 950 nm peaks. From this data, it is determined that absorption around 680 nm is due to both the D2 and D3 transitions, while the transition to the D1 state completely dominates at 950 nm.

**Table 3:** The TD-DFT vibronic absorption maximum peak wavelengths *λ* of the first two electronic transitions, and the vertical wavelengths *λ*, oscillator strengths *f*, and dominant orbital characters for the three lowest-energy calculated electronic transitions of the reduced PDI^–^ radical. The full set of the lowest ten transitions is given in Table S4

State	*λ*_vibronic_ [nm]	*λ* [nm]	*f*	Primary transition character	%	Secondary transition character	%
D1	974.4	844.3	0.032	βHOMO → LUMO	74	αLUMO → LUMO+1	26
D2	688.4	668.6	0.052	αLUMO → LUMO+2	97	—	—
D3	710.8	621.4	0.885	αLUMO → LUMO+1	72	βHOMO → LUMO	24

**Fig. 9:**
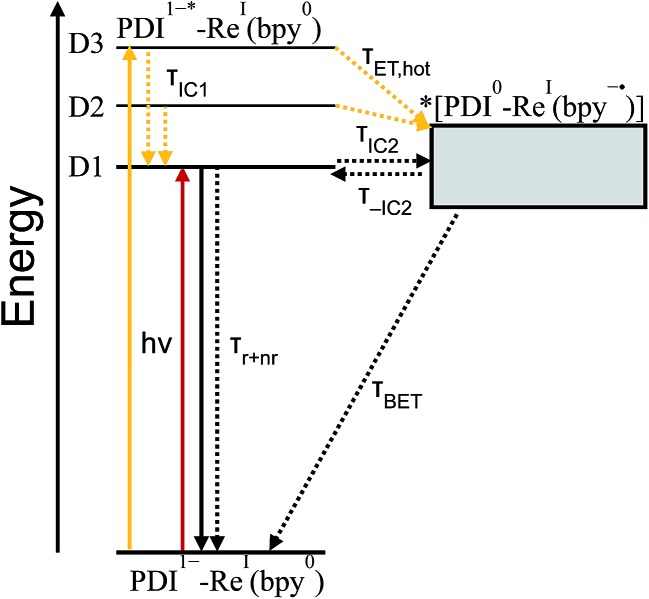
Jablonski diagram showing electron transfer in **PDI^1–^-Phbpy-Re-Py** after excitation at 950 nm (red) or 680 nm (yellow). Energy of the charge-transfer state is shown as a range to reflect the uncertainty of the exact energy of this state.

From the transition wavelengths and oscillator strengths shown in Table 3 and Fig. 8, it is apparent that excitation at 950 nm produces a vertically excited state of mostly HOMO → LUMO character which structurally relaxes to the optimized D1 excited state, while excitation at 680 nm produces a state with mostly LUMO → LUMO+1 character, but also some HOMO → LUMO and LUMO → LUMO+2 character. This vertical state predominantly relaxes to the optimized D3 excited state, but some optimized D1 and D2 is also expected. These states are shown in the energy-level diagram for the complex given in Fig. 9.

TD-DFT calculations reveal that the complex also has a CT excited state which we label as *[PDI^0^-Re(bpy˙^–^)], and which corresponds to direct charge-transfer excitation of the unpaired electron on the PDI^–^ to the empty LUMO on the bpy ligand. Due to extremely poor orbital overlap, this transition has negligible oscillator strength and is not predicted to contribute detectably to the ground-state absorption spectrum of **PDI^–^-Phbpy-Re-Py**. This CT state, shown as a gray box to reflect the uncertainty in its energy, is calculated to be approximately isoenergetic with the ^D1^PDI^–^*-Re(bpy) state, with which it is in equilibrium (see below).

As shown in Fig. 9, the D2 and D3 states relax *via* a set of processes that can be described as a combination of internal conversion to the D1 state, internal conversion to the *[PDI^0^-Re(bpy˙^–^)] state (collectively denoted *τ*_IC1_), and electron transfer to the *[PDI^0^-Re(bpy˙^–^)] state (*τ*_ET,hot_). It is likely that relaxation and electron transfer from the D3 state occurs within the instrument response, accounting for the immediate appearance of PDI^0^ bands in the *λ*_ex_ = 680 nm TA spectrum shown in Fig. 5D, while relaxation and electron transfer from the D2 state occurs with a lifetime of 1.8 ps, accounting for the evolution of the spectrum in the first several picoseconds (Fig. 5D, black trace).

As mentioned above, once the molecule reaches the D1 state, an equilibrium forms between the **PDI**-localized D1 state (which we can denote ^D1^PDI^–^*-Re(bpy)) and the *[PDI^0^-Re(bpy˙^–^)] CT state *via* a fast internal conversion mechanism (shown as *τ*_IC2_ and *τ*_–IC2_). This equilibrium accounts for the persistence of the PDI^–^* bands in the TA spectra as the PDI^0^ bands grow in, and the parallel decay of the excited-state and charge-shifted-state features. Because of the low oscillator strength of the transition to the *[PDI^0^-Re(bpy˙^–^)] state, this state does not decay radiatively on the picosecond timescale; instead this equilibrium decays *via* a combination of excited-state decay from the ^D1^PDI^–^*-Re(bpy) state (*τ*_*r*+*nr*_) and back-electron transfer from the *[PDI^0^-Re(bpy˙^–^)] state (*τ*_BET_), resulting in the observed lifetime of 52 ps.

Variable-temperature transient absorption experiments in the range 0–90 °C support the existence of the ^D1^PDI^–^*-Re(bpy) ⇌ *[PDI^0^-Re(bpy˙^–^)] equilibrium and suggest that the charge shift to the *[PDI^0^-Re(bpy˙^–^)] state is slightly uphill. Analysis of the SVD spectra for each set of TA data shows a dependence of the [PDI^0^] : [PDI^1–^*] ratio on temperature (see ESI† for details on the calculation of [PDI^0^] : [PDI^1–^*]). A plot of ln *K*_eq_ *vs.* 1/*T*, shown in Fig. S16,† gives a Δ*G* for the equilibrium of 0.21 ± 0.02 eV, supporting the existence of a ^D1^PDI^–^-Re ⇌ *[PDI^0^-Re(bpy˙^–^)] equilibrium that favors the *[PDI^0^-Re(bpy˙^–^)] state at higher temperature.

### Back electron transfer

**Fig. 10:**
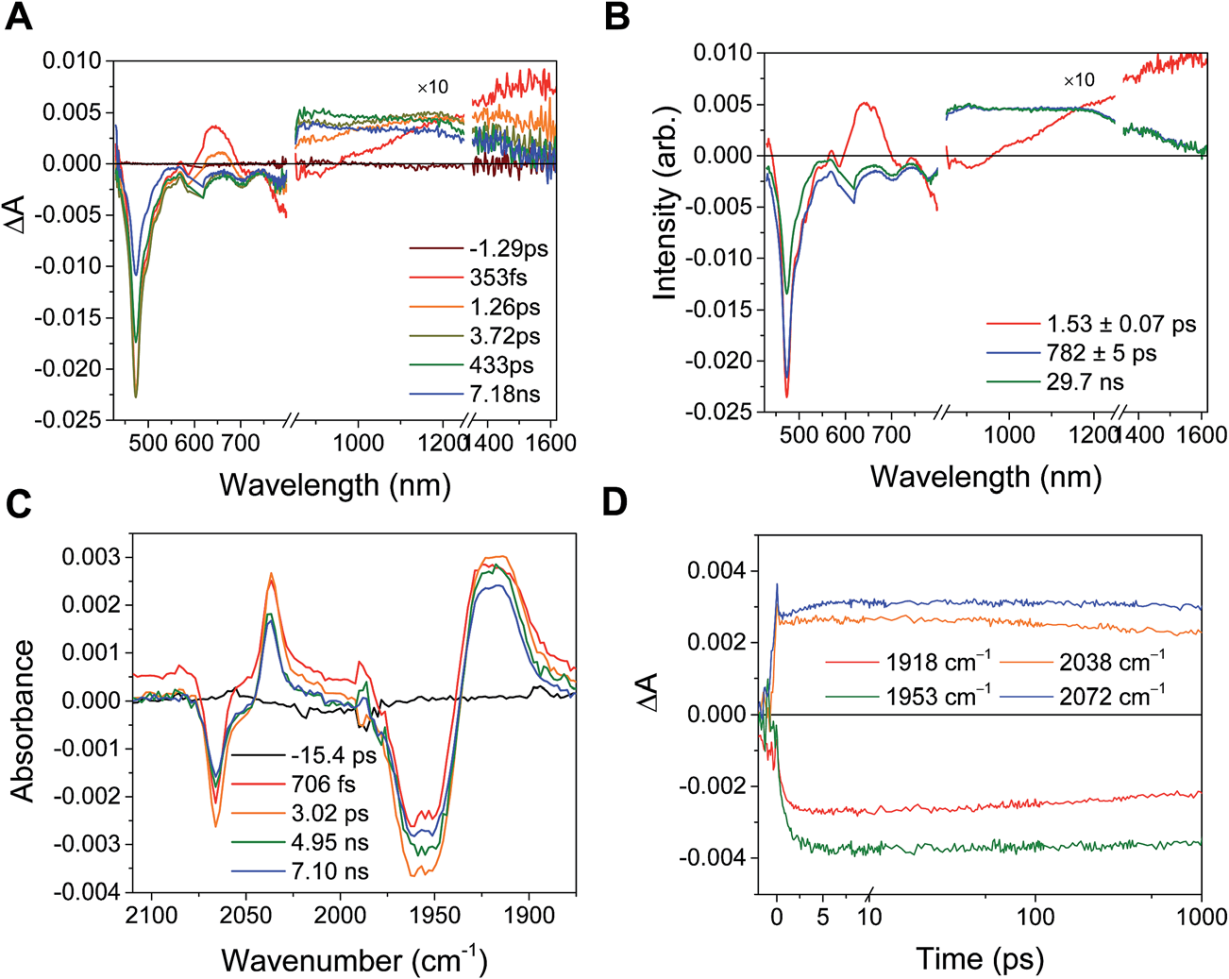
(A) Transient absorption and (B) species-associated spectra for complex **Phbpy-Re-PyPhNDI^1–^** (*λ*_ex_ = 605 nm) showing biphasic back electron transfer kinetics. Kinetic traces and fits are shown in Fig. S7.† (C) fsIR spectra and (D) kinetic traces with fits for complex **Phbpy-Re-PyPhNDI^1–^** (*λ*_ex_ = 605 nm) showing monophasic back electron transfer kinetics. The instrument response is approximately 2 ps.

In the **RDI*^n^*^–^-bpy-Re-py** complexes, and in **Phbpy-Re-PyPhPDI^1–^**, the final charge-shifted state exhibits straightforward single-exponential decay kinetics with the lifetimes shown in Table 2. For the complexes **Phbpy-Re-PyPhNDI^–^** and **Phbpy-Re-PyPhPDI^2–^** the charge-shifted state exhibits biexponential decay in the visible/NIR TA, but monoexponential decay in the fsIR, as shown in Fig. 10 and S11 and 12.†

Several trends in electron-transfer rate are apparent from the data. In all cases, attachment of the chromophore to the bipyridine ligand results in faster quenching than attachment of the same chromophore to a pyridine ligand on the Re center. It is also apparent that an increase in the driving force for electron transfer quenching for a given attachment motif generally results in an increase in electron transfer rate, although the rate for quenching of NDI^–^˙* ligated through bipyridine is slightly faster than quenching of PDI^2–^* with the same attachment motif. Similarly, the rate of back-electron transfer appears to correlate with the free energy change for that process, and back-electron transfer is orders of magnitude faster in the RDI-bipyridine-ligated complexes than in the RDI-pyridine-ligated complexes. This result is consistent with the fact that in the RDI-bipyridine-ligated complexes, the reduced bpy is separated from the oxidized chromophore by one phenyl spacer, whereas in the RDI-pyridine-ligated complexes the reduced bpy ligand is much further away from the oxidized chromophore.

## Conclusion

In the present report, we have described four complexes in which a reduced rylenediimide chromophore is oxidatively quenched by an appended Re(bpy)(CO)_3_ metal center, generating a reduced species. In some of our complexes, that charge separated state is observed to persist with a lifetime of tens or hundreds of nanoseconds. Femtosecond transient absorption data in the visible and near-infrared provided forward and back electron transfer rates. In studies of the mechanism of photocatalytic CO_2_ reduction by rhenium diimine complexes, reduction of the diimine ligand has been shown to be the step that initiates formation of a five-coordinate complex capable of binding CO_2_. This work demonstrates and elucidates this crucial initial photosensitization of these complexes. Analysis of the **PDI** radical anion excited states was aided by vibronically resolved TD-DFT computations to assign the electronic transitions in the NIR.

The bpy-linked complexes are similar to the metalloporphyrin–Re(bpy)(CO)_3_ complexes reported by Gabrielsson *et al.*,^11^ and separately by Ishitani and Inoue,^39^ in which oxidative quenching and charge recombination both occur on the timescale of single to tens of picoseconds. These complexes have been shown to photoreduce CO_2_ in the presence of sacrificial electron donors that coordinate to the metal center of the porphyrin.^20,35,39^ These results offer the possibility that our complexes could also be incorporated into catalytic systems under the proper conditions. Further research in our laboratory is directed at lengthening the lifetime of the charge-shifted state,^22^ investigating the photoelectrocatalytic properties of the complexes, and developing complexes with multiple photosensitizers that can accumulate multiple electrons on the catalytic center.
