# Supplementary material for: Photoinduced electron transfer from rylenediimide radical anions and dianions to Re(bpy)(CO)3 using red and near-infrared light
Source: Chem Sci. 2017 Feb 28;8(5):3821–31. doi: 10.1039/c6sc05103k (PMC5436599; doi:10.1039/c6sc05103k)
Supplement: Supplementary file 1 [file SC-008-C6SC05103K-s001.pdf]

Electronic Supplementary Material (ESI) for Chemical Science.
This journal is © The Royal Society of Chemistry 2017

Photosensitization of a CO_2 Reduction Catalyst with Red and Near-infrared Light using Rylenediimide Radical Anions and Dianions

Nathan T. La Porte,[a] Jose F. Martinez,[a] Svante Hedström,[b] Benjamin Rudshteyn,[b] Brian T. Phelan,[a] Catherine M. Mauck,[a] Ryan M . Young,[a] Victor S. Batista,[b] and Michael R. Wasielewski[a]

[a]Department of Chemistry and Argonne-Northwestern Solar Energy Research (ANSER) Center, Northwestern University, Evanston, Illinois 60208-3113, United States
[b]Department of Chemistry, Argonne-Northwestern Solar Energy Research (ANSER) Center, and Energy Sciences Institute. Yale University, New Haven, Connecticut, 06520, United States

NMR Spectroscopy

^{1}H NMR spectra were recorded on a Bruker Avance 500 TCI Cryoprobe Spectrometer. Chemical shifts are recorded in ppm (δ) in $CDCl_3$ (internal reference set to δ 7.26 ppm). ^{13}C NMR (126 MHz) spectra were recorded using a Bruker Avance III QNP Cryoprobe with simultaneous decoupling of ^{1}H nuclei and externally referenced to TMS set to 0 ppm. All spectra were recorded at 298 K.

Electronic absorbance spectroscopy

UV/Vis/NIR absorbance spectroscopy was performed on a Shimadzu UV-1601 spectrometer at 298 K.

Electrochemistry

Electrochemical measurements were performed using a CH Instruments Model 660A electrochemical workstation. All measurements were performed under argon using a 1.0 mm diameter platinum disk working electrode, a platinum wire counter electrode, a silver wire pseudoreference electrode, and 0.1 M tetrabutylammonium hexafluorophosphate in DMF. The ferrocene/ferrocenium redox couple (0.45 V vs SCE)[1] was used as an internal standard. $TBAPF_6$ was recrystallized twice from ethanol prior to use.

Calculation of Gibbs free energy for electron transfer reactions

The Gibbs free energy for the excited-state electron transfer reactions can be estimated using the following equation:

$$\Delta G_q = (E_{ox} - E_{red}) - E_{00} + \frac{Z_{RDI,p} Z_{bpy,p} e^2}{r_{RDI,bpy} \varepsilon_s} + \frac{Z_{RDI,p} Z_{Re,p} e^2}{r_{RDI,Re} \varepsilon_s} + \frac{Z_{Re,p} Z_{bpy,p} e^2}{r_{Re,bpy} \varepsilon_s} - \frac{Z_{RDI,r} Z_{bpy,r} e^2}{r_{RDI,bpy} \varepsilon_s} - \text{[illegible]}$$

where E_{ox} and E_{red} are the oxidation and reduction potentials of the donor and acceptor respectively, E_{00} is the energy of the RDI^{n-*} excited state[2], $Z_{RDI,p}$ and $Z_{bpy,p}$, are the charges on the RDI and bpy after electron transfer, $Z_{RDI,r}$, $Z_{bpy,r}$, $Z_{Re,r}$ are the charges on the RDI, bpy, and Re before electron transfer, e is the elementary charge, $r_{\mathrm{RDI,bpy}}$, $r_{\mathrm{RDI,Re}}$, and $r_{\mathrm{Re,bpy}}$ are the donor-acceptor distances (calculated from centroid to centroid using DFT-optimized geometries), and ε_s is the solvent dielectric constant.

The Gibbs free energy for the back electron transfer reactions (ΔG_{BET}) can be estimated using the following equation:

$$\Delta G_{ET} = (E_{ox} - E_{red}) + \frac{Z_{RDI,p}Z_{bpy,p}e^2}{r_{RDI,bpy}\varepsilon_s} + \frac{Z_{RDI,p}Z_{Re,p}e^2}{r_{RDI,Re}\varepsilon_s} + \frac{Z_{Re,p}Z_{bpy,p}e^2}{r_{Re,bpy}\varepsilon_s} - \frac{Z_{RDI,r}Z_{bpy,r}e^2}{r_{RDI,bpy}\varepsilon_s} - \frac{Z_{RDI,}\text{[illegible]}}{r_{R}\text{[illegible]}}$$

which is analogous to the previous equation but without the excited state energy.

Donor–acceptor distances calculated from optimized structures are shown in Table S1 below. Calculated geometries are included as supplementary information.

Table S1. Donor-acceptor distances in the complexes under study, calculated from the DFT-optimized geometries.

	$r_{RDI,bpy}$ (Å)	$r_{RDI,Re}$ (Å)	$r_{Re,bpy}$ (Å)
PDI-Phbpy-Re-Py	14.2	16.5	3.02
NDI-Phbpy-Re-Py	12.0	14.3	3.02
Phbpy-Re-PyPhPDI	16.6	16.5	3.02
Phbpy-Re-PyPhNDI	14.5	14.4	3.02

Computational Methodology

All density functional theory (DFT) calculations utilized the B3LYP functional,[3] as implemented in Gaussian 09, Revision D.01[4] software. The Def2SVP basis set (with corresponding pseudopotential on Re)[5] was used for geometry optimizations, and time-dependent DFT (TDDFT) calculations. The Def2TZVP basis set (pseudopotential on Re) was used for more accurate single-point energies as well as orbital analysis.[5] The Gaussian09 "ultrafine" numerical integration grid was used throughout, as well as the "tight" optimization criteria. All calculations were performed in the presence of a dielectric continuum of N,N−dimethylformamide (DMF) (ε = 37.219)[4] as described by the SMD model.[6-9] Vertical TDDFT calculations on the first 40 singlet excited states were performed.[10-16] The first three transitions in the PDI anion (D1, D2, D3) were subject to TDDFT excited-state optimizations and numerical frequency calculations, to create absorption spectra incorporating vibronic effects.[17] The D1 and D2 vibronic spectral lines were broadened with Gaussian functions with full-width half-maximum (FWHM) values of 270 cm^{-1}. For the D3 state, a single remaining imaginary frequency was projected out of the Hessian, and the D3 spectral lines used a broadening with FWHM=800 cm^{-1}. Alkyl and aryl groups on the PDI and NDI moieties, , were replaced with Me groups for computational expendiency. The D3 state consistently crossed over to the D2 or D1 state during optimization, so a slightly truncated structure with the methyl group replaced by hydrogen was used to optimize the D3 state.

The states of PDI vary from neutral singlet to anionic doublet to doubly anionic singlet upon two sequential reductions. The states of $[Re(Phbpy)(py)(CO)_3]^+$ (as well as the dyad) vary from cationic singlet to neutral doublet to anionic singlet upon two sequential reductions. Spin contamination in the cases of the doublets was found to be negligible using unrestricted DFT.

Treatment of Transient Absorption Data

Prior to kinetic analysis, the fsTA data were background/scatter-subtracted and chirp-corrected, and the visible and NIR data sets were spectrally merged (Surface Xplorer 4, Ultrafast Systems, LLC).

Kinetic Fitting of Transient Absorption Data

The kinetic analysis was performed using home written programs in MATLAB [18] and was based on a global fit to either selected single-wavelength kinetics or kinetic vectors following singular value decomposition (method is specified for each compound in Figures S2-S14). The time-resolution is given as w = 300 fs (full width at half maximum, FWHM); the assumption of a uniform instrument response across the frequency domain and a fixed time-zero (t_0) are implicit in global analysis.

Singular Value Decomposition

Factoring of the two-dimensional (signal vs time & frequency) data set by Singular Value Decomposition (SVD) is performed as implemented in the MATLAB software package.[18] This factoring produces an orthonormal set of basis spectra that describe the wavelength dependence of the species and a corresponding set of orthogonal vectors that describe the time-dependent amplitudes of the basis spectra.[19] These kinetic vectors are then fit using the global analysis method described below.

Multiple-Wavelength Global Fitting

The kinetic data from multiple different wavelengths are fit using the global analysis described below. Each wavelength is given an initial amplitude that is representative of the spectral intensity at time t_0, and varied independently to fit the data. The time/rate constants and t_0 are shared between the various kinetic data and are varied globally across the kinetic data in order to fit the species-associated model described below.

Species-Associated Fitting

We globally fit the dataset to a specified kinetic model and use the resultant populations to deconvolute the dataset and reconstruct species-associated spectra.

We use a first-order kinetic model with rate matrix K whose dimensions depend on the number of components in the model. For an A→B→(G)round model, used to fit all the data except the short-time data for **Phbpy-Re-PyPhNDI^{1-}** and **Phbpy-Re-PyPhPDI^{2-}**, the matrix is:

$$K = \begin{pmatrix} -k_{A\to B} & 0 \\ k_{A\to B} & -k_{B\to G} \end{pmatrix}$$

For the A→B+C→(G)round model used to fit the short-time data for **Phbpy-Re-PyPhNDI^{1-}** and **Phbpy-Re-PyPhPDI^{2-}**, the matrix is:

$$K = \begin{pmatrix} -k_{A\to B+C} & 0 & 0 \\ k_{A\to B+C} & -k_{B\to G} & 0 \\ k_{A\to B+C} & 0 & -k_{C\to G} \end{pmatrix}$$

The MATLAB program numerically the solves the differential equations through matrix methods,[20] then convolutes the solutions with a Gaussian instrument response function with width *w* (FWHM), before employing a least-squares fitting using a Levenberg-Marquardt or Simplex method to find the parameters which result in matches to the kinetic data.

Spectral Reconstruction
Once the fit parameters are established, they are fed directly into the differential equations, which were solved for the populations of the states in model—i.e., A(t), B(t) and C(t). Finally, the raw data matrix (with all the raw data) is deconvoluted with the populations as functions of time to produce the spectra associated with each species.

Determination of PDI^{1-*}–PDI^0 equilbrium constant

The extinction coefficient for PDI^{1-*} was determined by examination of the transient absorption spectra of PDI^{1-} (Figure 2 in main text). The known extinction coefficient for the bleach at 798 nm (ε = 49600 M^{-1} cm^{-1})[2] was used to determine the transient concentration of PDI^{1-*} at a number of time points. That concentration was then used to determine the extinction coefficient of PDI^{1-*} at 460 nm and 526 nm (ε_{460} = 25800 M^{-1} cm^{-1}, ε_{526} = 3300 M^{-1} cm^{-1}). The extinction coefficients of PDI^0 at those wavelengths were obtained from the literature[2] (ε_{460} = 19300 M^{-1} cm^{-1}, ε_{526} = 80000 M^{-1} cm^{-1}). The concentration of each species at a given timepoint can be expressed as the following system of equations:

$$A_{460} = [PDI^{1-*}] \times \varepsilon_{460,PDI^{1-*}} + [PDI^0] \times \varepsilon_{460,PDI^0}$$

$$A_{526} = [PDI^{1-*}] \times \varepsilon_{526,PDI^{1-*}} + [PDI^0] \times \varepsilon_{526,PDI^0}$$

This system can be solved to give the following expressions for $[PDI^{1-*}]$ and $[PDI^0]$:

$$[PDI^{1-*}] = \frac{A_{460} \times \varepsilon_{526,PDI^0} - A_{526} \times \varepsilon_{460,PDI^0}}{\varepsilon_{460,PDI^{1-*}} \times \varepsilon_{526,PDI^0} - \varepsilon_{526,PDI^0} \times \varepsilon_{460,PDI^0}}$$

$$[PDI^0] = \frac{A_{460} \times \varepsilon_{526,PDI^{1-*}} - A_{526} \times \varepsilon_{460,PDI^{1-*}}}{\varepsilon_{526,PDI^0} \times \varepsilon_{460,PDI^0} - \varepsilon_{460,PDI^{1-*}} \times \varepsilon_{526,PDI^0}}$$

These expressions were applied to the spectra derived from the SVD of the transient absorption data to derive the equilibrium constants for the $PDI^{1-*} \rightleftharpoons PDI^0$ excited-state electron transfer reaction.

Synthesis of compounds

C23-PDI-PhBr and **C23-PDI-PhI** were synthesized using a modification of a literature procedure.[21] 12-aminotricosane was synthesized according to a literature procedure.[22] 4-bromobipyridine was synthesized by a modification of literature procedures.[23, 24]

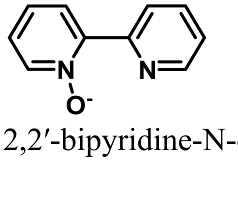

2,2′-bipyridine-N-oxide

2,2-Bipyridine (6.00 g, 38.44 mmol) was dissolved in 100 mL of trifluoroacetic acid under air. 30 mass % hydrogen peroxide solution (12 mL) was added and the mixture obtained was stirred at ambient temperature for 5 h. Then 5M NaOH (100 mL) was slowly added to the solution and allowed to stir for 1hr. The solution was mixed with dichloromethane (300 mL) and washed with 5M NaOH (3 x 150 mL). Water phase was reextracted twice with dichloromethane (2 x 100 mL) and the combined organic phases dried with $MgSO_4$, filtered, and dried under reduced pressure. The product was obtained as a pale yellow oil which crystalized after overnight into an off-white solid (6.16 g, 93%). **^{1}H NMR** (500 MHz, Chloroform-*d*) δ 8.89 (dt, *J* = 4.6, 1.3 Hz, 1H), 8.71 (dd, *J* = 8.1, 1.1 Hz, 1H), 8.29 (dd, *J* = 6.6, 1.2 Hz, 1H), 8.16 (dd, *J* = 8.1, 2.2 Hz, 1H), 7.81 (td, *J* = 7.8, 1.9 Hz, 1H), 7.36 – 7.30 (m, 2H), 7.27 – 7.23 (m, 1H).

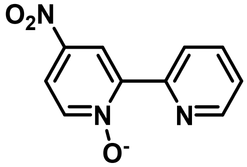

4-nitro-2,2′-bipyridine-N-oxide

2,2′-bipyridine-N-oxide (6.10 g, 35.46 mmol) and potassium nitrate (19.60 g, 193.90 mmol) in concentrated sulfuric acid (50 mL) was stirred at 80 °C for 30 hours. Then, the mixture was poured onto ice (100 g) and neutralized with 25 mass % NaOH to pH 9.0. The precipitate was filtered off, washed with cold water and dried under a high vacuum at 100 °C. An off-white solid was obtained (2.31 g, 30%). **^{1}H NMR** (500 MHz, Chloroform-*d*) δ 9.17 (d, *J* = 3.3 Hz, 1H), 8.89 (dt, *J* = 8.1, 1.1 Hz, 1H), 8.79 (ddd, *J* = 4.7, 1.9, 1.0 Hz, 1H), 8.36 (d, *J* = 7.2 Hz, 1H), 8.06 (dd, *J* = 7.2, 3.3 Hz, 1H), 7.88 (td, *J* = 7.9, 1.8 Hz, 1H), 7.43 (ddd, *J* = 7.6, 4.7, 1.2 Hz, 1H).

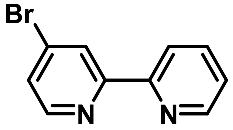

4-bromo-2,2′-bipyridine

4-nitro-2,2′-bipyridine-N-oxide (1.0 g, 4.6 mmol) was dissolved in glacial acetic acid (20 mL). While stirrin g, acetyl bromide was added. After a short while, a yellow precipitate was formed. At this time, phosphorous tribromide (5 mL) was added to the resulting suspension and heated to 40 °C for 15 minutes. The solution was then heated to reflux under air for 1 hr, whereby a new incredibly viscous precipitate formed. After cooling to room temperature, the solution was decanted and the remaining sticky residue was dissolved in water. The acid solution was neutralized to pH 9 with concentrated NaOH and filtered to obtain a yellow solid. The crude product was purified further by sublimation to yield an off-white solid (72%, 0.8 g). **^{1}H NMR** (500 MHz, Chloroform-*d*) δ 8.69 (ddd, *J* = 4.8, 1.8, 0.9 Hz, 1H), 8.63 (d, *J* = 1.9, Hz, 1H), 8.49 (d, *J* = 5.2, 1H), 8.39 (dt, *J* = 8.0, 1.1 Hz, 1H), 7.83 (td, *J* = 7.7, 1.8 Hz, 1H), 7.48 (dd, *J* = 5.2, 2.0 Hz, 1H), 7.34 (ddd, *J* = 7.5, 4.8, 1.2 Hz, 1H).

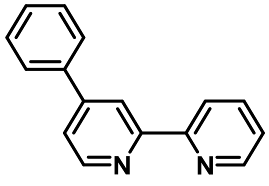

4-phenyl-2,2′-bipyridine

In an oven dried round bottom flask with a magnetic stirrer and reflux condenser, Phenylboronic acid (0.187, 1.534mole), 4-Bromo-2,2′-bipyridine (0.30 g, 1.28 mmol), and Na_2CO_3 (1.02 g, 9.60 mmol) were dissolved in THF (50 mL) and H_2O (15 mL) and put under N_2. After 15 minutes of purgin g, fresh Pd[PPh3]4 (0.07 g, 5% eq) was added via the second port and purged for 5 minutes. The solution was set to reflux overnight. The solution was cooled to room temperature and the solvent was removed under reduced pressure. The crude material was dissolved in dichloromethane and ran through a celite plug. The solvent was removed under reduced pressure. The compound was not purified further and a conversion of 100% was assumed for further purposes.

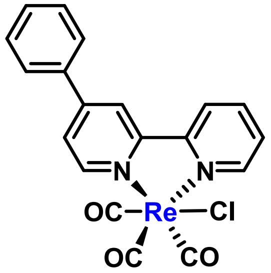

Phby-Re-Cl

In a nitrogen filled glove box, an oven dried pressure flask with a magnetic stirrer was filled with 4-phenyl-2,2′-bipyridine (0.30 g, 1.29 mmol), $Re(CO)_5Cl$ (0.55 g, 1.55 mmol), and toluene (30 mL). The pressure flask was capped and brought out of the glovebox and heated to 80 °C overnight. The solution was cooled to room temperature and the solvent was removed by reduced pressure. The solid was then column chromatographed on silica using a gradient between dichloromethane and Acetone (100 → 98:2). The compound was then dissolved in minimal dichloromethane and layered with diethyl ether. Upon filtering, a bright yellow compound was obtained (0.46 g, 66%). **^{1}H NMR** (500 MHz, Chloroform-*d*) δ 9.10 (ddd, *J* = 5.5, 1.6, 0.8 Hz, 1H), 9.07 (d, *J* = 5.8 Hz, 1H), 8.32 (d, *J* = 1.8 Hz, 1H), 8.30 (dt, *J* = 8.1, 1.0 Hz, 1H), 8.09 (td, *J* = 7.9, 1.6 Hz, 1H), 7.72 – 7.68 (m, 3H), 7.62 – 7.54 (m, 4H).

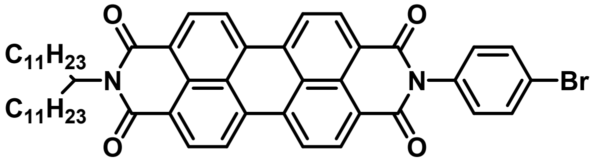

C23PDI-PhBr

1.40 g (3.57 mmol) perylene-3,4,9,10-dianhydride (PDA) and 736 mg (4.28 mmol) 4-bromoaniline were combined with 19 g imidazole in a 50 mL roundbottom flask. The flask was purged for 45 minutes with N_2 and 1.9 mL (1.47 g, 4.35 mmol) 12-aminotricosane that had been melted over a steam bath was added via syringe. The flask was placed in a preheated 130° oil bath and stirred for 2 hours. The flask was then cooled to room temperature and the contents suspended in 125 mL dichloromethane with the aid of sonication and washed with 100 mL 2 M HCl. The emulsion was broken with 50 mL isopropanol and the aqueous layer was washed twice with 100 mL dichloromethane. The organic layers were combined, dried over $MgSO_4$, gravity filtered and stripped. **C23PDI-PhBr** was separated from the symmetric **$C23_2$-PDI** on a silica column with 80-100% DCM:hexanes (desired product eluted second). Yield: 1.42 g (38%) **^{1}H NMR** (500 MHz, Chloroform-*d*) δ 8.53 (s, 2H), 8.48 (d, *J* = 7.9 Hz, 4H), 8.28 (dd, *J* = 8.1, 5.8 Hz, 4H), 7.63 (d, *J* = 8.3 Hz, 2H), 7.23 (d, *J* = 8.5 Hz, 2H), 5.44 – 4.77 (m, 1H), 2.18 (dtd, *J* =

14.1, 9.5, 4.7 Hz, 2H), 1.86 (ddt, J = 14.7, 11.1, 5.4 Hz, 2H), 1.29 (tt, J = 13.2, 5.9 Hz, 8H), 1.15 (d, J = 10.3 Hz, 36H), 0.78 (t, J = 6.9 Hz, 8H).

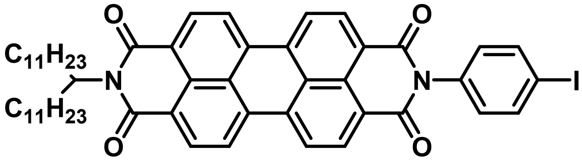

C23PDI-PhI

1.40 g (3.57 mmol) perylene-3,4,9,10-dianhydride (PDA) and 937 mg (4.28 mmol) 4-iodoaniline were combined with 19 g imidazole in a 50 mL roundbottom flask. The flask was purged for 45 minutes with N_2 and 1.9 mL (1.47 g, 4.35 mmol) 12-aminotricosane that had been melted over a steam bath was added via syringe. The flask was placed in a preheated 130° oil bath and stirred for 2 hours. The flask was then cooled to room temperature and the contents suspended in 125 mL dichloromethane with the aid of sonication and washed with 100 mL 2 M HCl. The aqueous layer was washed twice with 125 mL dichloromethane. The organic layers were combined, dried over Na_2CO_3, gravity filtered and dried under vacuum. C23PDI-PhI was separated from the symmetric C23$_2$-PDI on a silica column with 80-100% DCM:hexanes followed by 0-8% ethyl acetate:DCM (desired product eluted second). Yield: 674 mg (17%). **^{1}H NMR** (500 MHz, Chloroform-*d*) δ 8.70 (d, J = 8.0 Hz, 1H), 8.67 – 8.58 (d, J = 8.1 Hz, 2H), 7.88 (d, J = 8.4 Hz, 2H), 7.10 (d, J = 8.4 Hz, 2H), 5.16 (m, J =, 1H), 2.23 (dtd, J = 14.2, 9.6, 4.8 Hz, 2H), 1.85 (ddt, J = 14.7, 10.7, 4.8 Hz, 2H), 1.42-1.12 (m, 36H), 0.82 (t, J = 6.9 Hz, 6H).

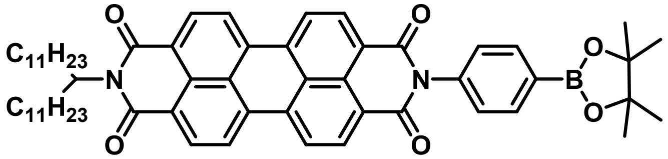

C23PDI-PhBpin

520 mg (0.6 mmol) **C23PDI-PhBr** and 315 mg (1.2 mmol) bispinacolatoboron (B_2pin_2) or 500 mg (0.54 mmol) **C23PDI-PhI** and 285 mg (1.1 mmol) B_2pin_2 were combined with 180 mg potassium acetate and 45 mg (0.06 mmol) Pd(dppf)Cl_2 in a 100 mL two-neck roundbottom flask fitted with a condensor. The flask was evacuated and backfilled three times with N_2. 50 mL dry, degassed DMF was injected and the flask was stirred at 100° overnight (C23PDI-PhBr) or for one hour (C23PDI-PhI). The flask was cooled to room temperature, the contents diluted with 50 mL DCM, and washed with 3x100 mL DI H_2O. The organic layer was concentrated to 3 mL and 50 mL MeOH was added to precipitate the product, which was collected by centrifugation, washed with an additional 50 mL MeOH and dried. Yield: 529 mg (from **C23PDI-PhBr**, 96%), 430 mg (from **C23PDI-PhI**, 87%). **^{1}H NMR** (500 MHz, Chloroform-*d*) δ 8.68 (m, 8H), 8.01 (d, J = 8.0 Hz, 2H), 7.35 (d, J = 8.0 Hz, 2H), 5.25-5.06 (m, 1H), 2.23 (m, 2H), 1.84 (m, 2H), 1.36 (s, 12H), 1.42-1.10 (m, 36H), 0.82 (t, J = 6.9 Hz, 6H).

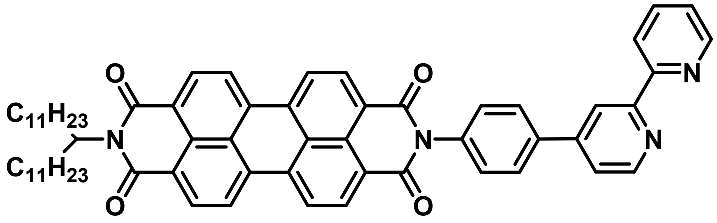

C23PDI-Phbpy

425 mg (0.33 mmol) **C23PDI-PhBpin**, 81 mg (0.33 mmol) 4-bromobipyridine, and 361 mg (3.33 mmol) were combined in 40 mL THF and 10 mL DI H_2O in a two-neck 100 mL roundbottom flask and sparged 30 minutes with N_2. 52 mg (0.033 mmol) $Pd(PPh_3)_4$ (Strem) was added under flow of N_2 and the solution sparged for an additional ten minutes, then refluxed overnight. The flask was cooled to room temperature, the contents diluted with 300 mL DCM, washed with 3x200 mL DI H_2O, gravity filtered and dried under vacuum. The precipitate was dissolved in minimal DCM and 50 mL MeOH added to precipitate product. The product was collected by centrifugation, washed with an additional 50 mL MeOH and dried. Yield: 287 mg (91%). **^{1}H NMR** (500 MHz, Chloroform-*d*) δ 8.76-8.61 (m, 8 H), 8.75 (d, *J* = 8.1 Hz, 1H), 8.71 (d, *J* = 4.6 Hz, 1H), 8.66 (s, 1H), 8.45 (d, *J* = 8.0, 1H), 7.96 (d, *J* = 8.4 Hz, 2H), 7.84 (td, *J* = 7.7, 1.8 Hz, 1H), 7.60 (dd, *J* = 5.0, 1.6 Hz, 1H), 7.50 (d, *J* = 8.3 Hz, 2H), 7.33 (dd, *J* = 7.7, 4.6 Hz, 1H), 5.17 (tt, *J* = 9.5, 5.7 Hz 1H), 2.23 (m, 2H), 1.84 (m, 2H), 1.42-1.10 (m, 36H), 0.82 (t, *J* = 6.9 Hz, 6H).

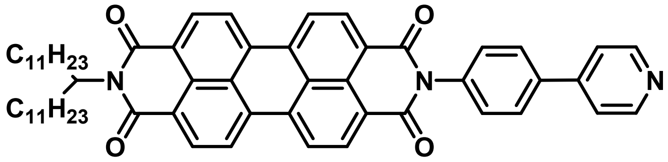

C23PDI-PhPy

128 mg (0.10 mmol) **C23PDI-PhBpin**, 21.4 mg (0.11 mmol) 4-bromopyridine HCl, and 108 mg (1.0 mmol) Na_2CO_3 were combined in 20 mL THF and 5 mL DI H_2O in a 50 mL two-necked roundbottom flask and sparged 30 minutes with N_2. 16 mg (0.01 mmol) $Pd(PPh_3)_4$ was added under flow of N_3 and the solution sparged for an additional ten minutes, then refluxed overnight. A great deal of insoluble red product forms on the inside of the flask, likely trapping product within it. The flask was cooled to room temperature, diluted with 50 mL DCM, washed with 50 mL saturated KOH and 3x100 mL DI H_2O and dried under vacuum. The precipitate was dissolved in minimal DCM and 50 mL MeOH was added to precipitate product. The suspension was filtered through Celite and the precipitate washed with MeOH, then washed off the Celite with DCM. The solution was collected and dried under vacuum. Yield: 15 mg (17%).

C23PDI-PhPy was also prepared from **C23PDI-PhBr** by the following method: In an oven dried round bottom flask with a magnetic stirrer and reflux condenser, **C23PDI-PhBr** (0.10 g, 0.12 mmol), 4-Pyridinylboronic acid (0.03 g, 0.23 mmol), and Na_2CO_3 (0.70 g, 6.60 mmol) were dissolved in THF (150 mL) and H_2O (30 mL) and put under N_2. After 15 minutes of purging, fresh $Pd(PPh_3)_4$ (0.007 g, 5% eq) was added via the second port and purged for 5 minutes. The solution was set to reflux overnight. The solution was cooled to room temperature and the solvent was removed under reduced pressure. The remaining solid was suspended in MeOH and filtered through a celite plug. Dichloromethane was then used to elute the product. After the

solvent was removed under reduced pressure a dark red solid was obtained (40%, 0.04g). **^{1}H NMR** (500 MHz, Chloroform-*d*) δ 8.73 (d, *J* = 10.0 Hz, 2H), 8.71 – 8.66 (m, 8H), 7.83 – 7.81 (m, 2H), 7.60 – 7.57 (m, 2H), 7.52 – 7.48 (s, 2H), 5.14 – 5.08 (m, 1H), 2.25-2.15 (m, 2H), 1.87-1.83 (m, 2H), 1.23-1.17 (m, 36H), 0.82 (t, *J* = 7.5 Hz, 6H)

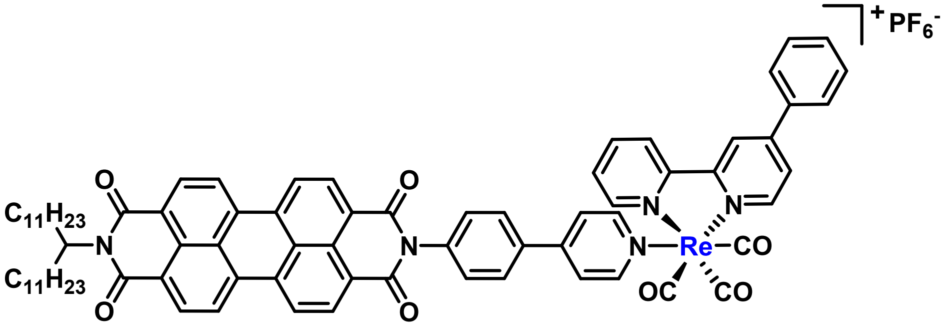

Phbpy-Re-PyPhPDI

In a nitrogen filled glove box, an oven dried pressure flask with a magnetic stirrer was filled with **C23PDI-PhPy** (0.04 g, 0.046 mmol), **Phby-Re-Cl** (0.03 g, 0.055 mmol), $AgPF_6$ (0.015 mg, 0.059 mmol), and dichloromethane (40 mL). The pressure flask was capped and brought out of the glovebox and heated to 80 °C overnight. The solution was cooled to room temperature and filtered through Celite. The solvent was removed under reduced pressure and then column chromatographed on silica using a gradient between dichloromethane and Acetone (100 → 96:4 → 90:10 → 80:20). A dark red compound was obtained (0.03 g, 14%). **^{1}H NMR** (500 MHz, Chloroform-*d*) δ 9.10 – 9.02 (m, 2H), 8.74 (d, *J* = 8.3 Hz, 1H), 8.70 (d, *J* = 1.9 Hz, 1H), 8.69 – 8.55 (m, 8H), 8.36 (td, *J* = 8.0, 1.8 Hz, 1H), 8.24 – 8.19 (m, 2H), 7.90 – 7.85 (m, 3H), 7.76 – 7.70 (m, 3H), 7.64 – 7.60 (m, 2H), 7.60 – 7.52 (m, 3H), 7.44 – 7.40 (m, 2H), 5.17-5.12 (m, 1H), 2.25-2.15 (m, 2H), 1.87-1.83 (m, 2H), 1.20-1.17 (m, 36H), 0.82 (t, 6H) **^{13}C NMR** (126 MHz, $CDCl_3$) δ 163.4, 157.5, 156.3, 155.9, 153.8, 152.8, 152.7, 152.0, 151.1, 141.8, 137.4, 135.9, 135.4, 135.0, 134.2, 132.0, 131.5, 130.0, 129.9, 129.6, 128.9, 128.4, 127.8, 126.8, 126.5, 126.3, 126.1, 124.9, 123.5, 123.3, 123.2, 123.0, 105.3, 55.0, 32.5, 32.0, 29.8, 29.7, 29.7, 29.6, 29.4, 27.1, 22.8, 14.2

HRMS-ESI (*m/z*): calculated $C_{77}H_{75}N_5O_7Re$ $[M - PF_6]^+$: 1368.5224, found 1368.5217

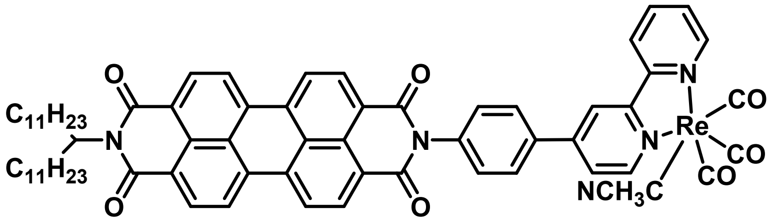

PDI-Phbpy-Re-ACN

68 mg $Re(CO)_5Br$ and 188 mg **C23PDI-Phbpy** were combined in 50 mL toluene in a 100 mL pressure tube in the glovebox and heated to 110° for 90 minutes. Tolene was removed under vacuum and the precipitate was dissolved in 20 mL DCM. 4 mL acetonitrile and 56 mg $AgPF_6$ were added and the solution was heated in the pressure tube at 55° overnight. The solvent was removed under vacuum and the precipitate dissolved in 100 mL acetonitrile, filtered through Celite, and dried under vacuum. Yield: 198 mg (50%). **^{1}H NMR** (500 MHz, Chloroform-*d*) δ 8.95 (t, *J* = 6.5 Hz, 2H), 8.75-8.58 (m, 10H), 8.27 (td, *J* = 8.0, 1.1 Hz, 1H), 8.06 (d, *J* = 8.2 Hz, 2H), 7.83 (dd, *J* = 5.7, 1.1 Hz, 1H), 7.65 (t, *J* = 6.0 Hz, 1H), 7.62 (d, *J* = 8.1 Hz, 2H), 5.16 (m,

1H), 2.23 (m, 2H), 2.20 (s, 3H), 1.84 (m, 2H), 1.36 (s, 12H), 1.42-1.10 (m, 36H), 0.82 (t, J = 6.9 Hz, 6H)

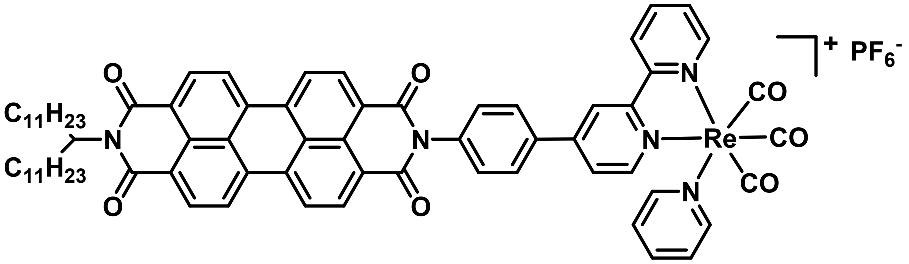

PDI-Phbpy-Re-Py

45 mg **PDI-Phbpy-Re-ACN** and 50 µL pyridine were dissolved in 25 mL chloroform and refluxed overnight. Solvent was removed under vacuum. Product was chromatographed on silica with 0-5% MeOH:DCM. The main band was collected and solvent evaporated under vacuum. Precipitate was dissolved in DCM and poured over a silica plug. The red material that remained at the top of the plug was exhaustively washed with DCM until eluent was colorless. The layer of silica containing red material was scraped off the top of the plug and extracted with 10% MeOH:DCM. The solvent was evaporated under vacuum to produce pure product. **^{1}H NMR** (500 MHz, Chloroform-*d*) δ 9.06 (t, J = 5.0 Hz, 2H), 8.81-8.60 (m, 10H), 8.34 (m, 1H), 8.17 (d, J = 5.1 Hz, 2H), 8.08 (d, J = 7.9 Hz, 2H), 7.91 (d, J = 5.7 Hz, 1H), 7.83 (t, J = 7.3 Hz, 1H), 7.73 (t, J = 6.5 Hz, 1H), 7.60 (d, J = 7.3 Hz, 2H), 7.40 (m, 2H), 5.16 (m, 1H), 2.23 (m, 2H), 1.84 (m, 2H), 1.36 (s, 12H), 1.42-1.10 (m, 36H), 0.82 (t, J = 6.9 Hz, 6H) **^{13}C NMR** (126 MHz, Chloroform-*d*) δ 195.69, 190.93, 163.24, 156.23, 155.76, 152.93, 152.58, 151.63, 141.63, 139.94, 137.92, 135.41, 135.15, 133.91, 131.78, 130.28, 129.62, 129.32, 128.86, 128.75, 127.24, 126.41, 126.21, 125.97, 123.35, 123.02, 122.79, 54.88, 32.36, 31.91, 29.64, 29.62, 29.58, 29.34, 27.06, 22.67, 14.11.

HRMS-ESI (*m/z*): calculated $C_{71}H_{71}N_5O_7Re$ $[M - PF_6]^+$: 1292.4911, found 1292.4900

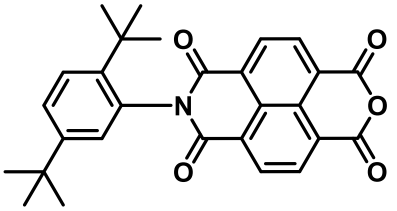

DtB-NIA

In an oven dried round bottom flask with a magnetic stirrer, reflux condenser, and addition funnel. Naphthalene-1,4,5,8-tetracarboxylic dianhydride (13.08 g, 48.78 mmol) was added to the flask with pyridine (200 mL) and DMF (200 mL) and heated to reflux 120 °C under N_2. 2,5-Di-tert-butylaniline (5.0 g, 24.39) was dissolved in pyridine (50 mL) and added via the addition funnel and slowly added over a 30-minute period. The reaction was allowed to go overnight. The solution was cooled to room temperature and the solvent was removed under reduced pressure. The product was then extracted with dichloromethane (200 mL) and washed with water (3 x 100 mL). The organic layer was separated and the solvent was removed under reduced pressure. The remaining solid was purified by column chromatography using dichloromethane and dichloromethane:Acetone (99:1) to obtain an off-white solid (2.50 g, 23%). **^{1}H NMR** (500 MHz, Chloroform-*d*) δ 8.91 – 8.83 (m, 4H), 7.61 (d, J = 8.6 Hz, 1H), 7.50 (dd, J = 8.6, 2.2 Hz, 1H), 6.98 (d, J = 2.1 Hz, 1H), 1.34 (s, 9H), 1.26 (d, 9H).

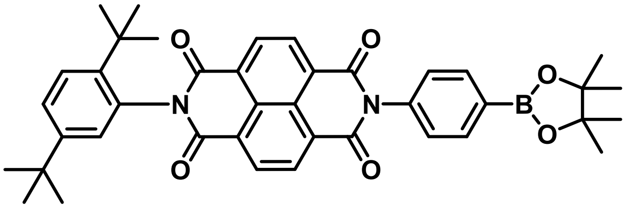

DtB-NDI-PhBpin

In an oven dried round bottom flask with a magnetic stirrer and reflux condenser, **DtB-NIA** (1.50 g, 3.29 mmol) and 4-(tetramethyl-1,3,2-dioxaborolan-2-yl)aniline (1.02 g, 4.65 mmol) were dissolved in pyridine (50 mL) and heated to 120 °C under N_2 overnight. The solution was cooled to room temperature and pyridine was removed in vacuo. The gooey material was then dissolved in 30 mL of dichloromethane and washed with 30 mL of 1M HCl. The organic layer was separated and the solvent was removed under reduced pressure. The remaining solid was washed with 20 mL MeOH, affording an off-white colored powder (1.50 g, 70%). **^{1}H NMR** (500 MHz, Chloroform-*d*) δ 8.85 (s, 4H), 8.04 (d, *J* = 8.1 Hz, 2H), 7.61 (d, *J* = 8.6 Hz, 1H), 7.49 (dd, *J* = 8.6, 2.2 Hz, 1H), 7.37 – 7.32 (m, 2H), 7.01 (d, *J* = 2.2 Hz, 1H), 1.38 (s, 12H), 1.33 (s, 9H), 1.28 (s, 9H).

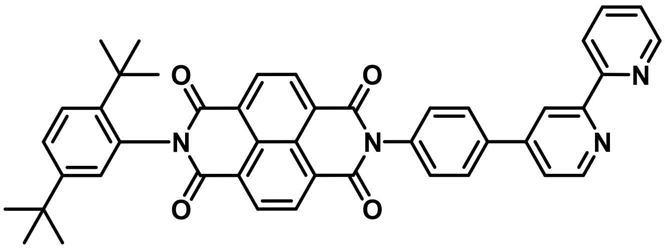

DtB-NDI-Phbpy

In an oven dried round bottom flask with a magnetic stirrer and reflux condenser, compound **N2** (0.10 g, 0.15 mmol), **B3** (0.04 g, 0.18 mmol), and Na_2CO_3 (0.127 g, 1.2 mmol) were dissolved in THF (33mL) and H_2O (7mL) and put under N_2. After 15 minutes of purging, fresh Pd[PPh3]4 (8.7mg , 5% eq) was added via the second port and purged for 5 minutes. The solution was set to reflux overnight. The solution was cooled to room temperature and the solvent was removed under reduced pressure. The crude material was then dissolved in dichloromethane and ran through a celite plug. The solvent was removed under reduced pressure. The remaining solid was sonicated in minimal MeOH and allowed to sit overnight in a freezer. The solution was filtered and the light brown solid was collected (20%, 0.043g). **^{1}H NMR** (500 MHz, Chloroform-*d*)) δ 8.89 (s, 4H), 8.80 – 8.71 (m, 3H), 8.48 (dt, *J* = 8.0, 1.2 Hz, 1H), 7.99 (d, *J* = 8.3 Hz, 2H), 7.86 (td, *J* = 7.8, 1.8 Hz, 1H), 7.64 – 7.59 (m, 2H), 7.52 – 7.48 (m, 3H), 7.38 – 7.34 (m, 1H), 7.03 (d, *J* = 2.2 Hz, 1H), 1.34 (s, 9H), 1.29 (s, 9H).

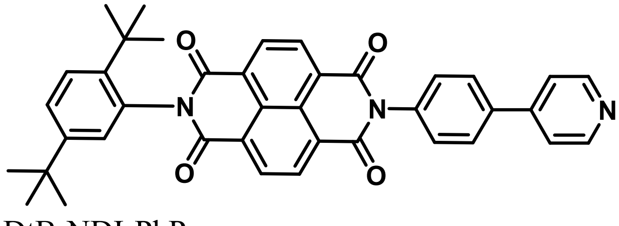

DtB-NDI-PhPy

In an oven dried round bottom flask with a magnetic stirrer and reflux condenser, **DtB-NDI-PhBpin** (0.50 g, 0.15 mmol), 4-Bromopyridine hydrochloride (0.22 g, 1.13 mmol), and Na_2CO_3

(0.70 g, 6.60 mmol) were dissolved in THF (150 mL) and H_2O (30 mL) and put under N_2. After 15 minutes of purgin g, fresh Pd[PPh3]4 (0.06 g, 5% eq) was added via the second port and purged for 5 minutes. The solution was set to reflux overnight. The solution was cooled to room temperature and the solvent was removed under reduced pressure. The remaining solid was put through a silica plug. Using dichloromethane, and ultimately dichloromethane:MeOH (90:10) to elute the product. The solid was then sonicated in minimal MeOH and filtered to obtain an off-white solid (49%, 0.043g). **^{1}H NMR** (500 MHz, Chloroform-*d*) δ 8.86 (d, 4H), 8.71 (d, 2H), 7.85-7.80 (d, 2H), 7.62-7.53 (m, 3H), 7.50-7.43 (m, 3H), 7.02 (s, 1H), 1.32 (s, 9H), 1.27 (s, 9H).

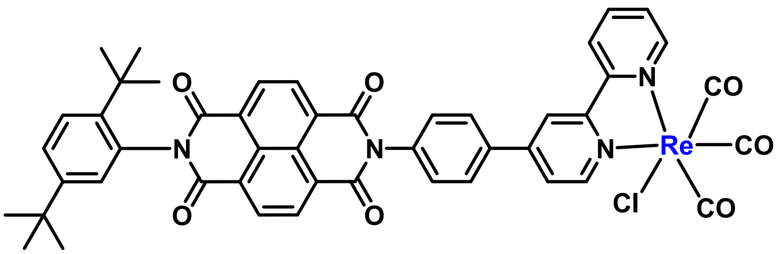

NDI-Phbpy-Re-Cl

In a nitrogen filled glove box, an oven dried pressure flask with a magnetic stirrer was filled with compound **N3** (0.1 g, 0.15 mmol), pentacarbonylchlororhenium(I) (0.065 g, 0.18 mmol), and dichloromethane (30 mL). The pressure flask was capped and brought out of the glovebox and heated to 80 °C overnight. The solution was cooled to room temperature and the solvent was removed by reduced pressure. The solid was then column chromatographed on silica using a gradient between dichloromethane and Ethyl Acetate (100 → 98:2 → 85:15). The compound was then dissolved in minimal dichloromethane and layered with diethyl ether. Upon filtering, a bright yellow compound was obtained with sufficient purity to carry through to the next step. Yield: (0.03 g, 20%). **^{1}H NMR** (500 MHz, Chloroform-*d*) δ 9.12 (td, J = 5.6, 1.1 Hz, 2H), 8.90 (s, 4H), 8.38 (d, J = 1.8 Hz, 1H), 8.35 – 8.30 (m, 1H), 8.10 (td, J = 7.6, 3.7 Hz, 1H), 7.93 – 7.87 (m, 2H), 7.75 (dt, J = 5.9, 1.9 Hz, 1H), 7.65 – 7.58 (m, 2H), 7.58 – 7.54 (m, 2H), 7.51 (dd, J = 8.6, 2.2 Hz, 1H), 7.03 (d, J = 2.2 Hz, 1H), 1.34 (s, 9H), 1.29 (s, 9H).

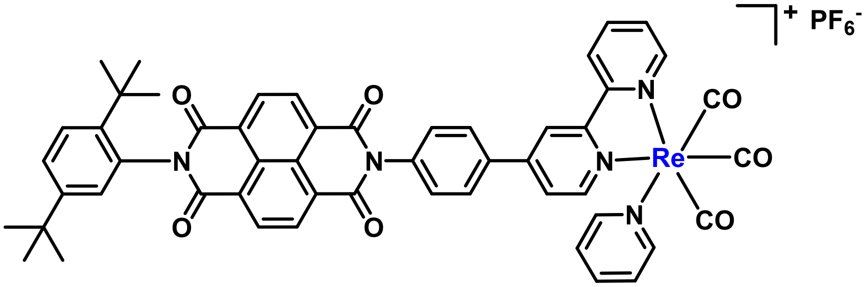

NDI-Phbpy-Re-Py

In a nitrogen filled glove box, an oven dried pressure flask with a magnetic stirrer was filled with **NDI-Phbpy-Re-Cl** (30 mg, 0.030 mmol), $AgPF_6$ (8 mg, 0.033 mmol), pyridine (10µL, 0.036), and dichloromethane (40 mL). The pressure flask was capped and brought out of the glovebox and heated to 80 °C overnight. The solution was cooled to room temperature and filtered through celite. The solvent was removed under reduced pressure and then column chromatographed on silica using a gradient between dichloromethane and Acetone (100 → 90:10). The compound was then dissolved in minimal dichloromethane and layered with diethyl ether. Upon filtering, a bright yellow compound was obtained (3 mg, 8%). **^{1}H NMR** (500 MHz, Chloroform-*d*) δ 9.10 –

9.03 (m, 2H), 8.86 (s, 4H), 8.77 – 8.69 (m, 2H), 8.32 (td, *J* = 7.9, 1.5 Hz, 1H), 8.16 (dt, *J* = 5.2, 1.5 Hz, 2H), 8.10 (d, *J* = 8.5 Hz, 2H), 7.91 (dd, *J* = 5.9, 1.9 Hz, 1H), 7.82 (tt, *J* = 7.7, 1.5 Hz, 1H), 7.72 (ddd, *J* = 7.4, 5.6, 1.1 Hz, 1H), 7.63 – 7.54 (m, 3H), 7.48 (dd, *J* = 8.6, 2.2 Hz, 1H), 7.42 – 7.35 (m, 2H), 7.01 (d, *J* = 2.2 Hz, 1H), 1.32 (s, 9H), 1.27 (s, 9H) **^{13}C NMR** (126 MHz, $CDCl_3$) δ 163.9, 162.9, 156.5, 155.9, 152.8, 152.8, 152.6, 151.7, 150.5, 143.8, 141.8, 140.1, 137.5, 136.0, 132.1, 131.7, 131.6, 130.3, 129.1, 129.1, 128.9, 127.6, 127.5, 127.5, 127.4, 126.9, 126.4, 126.2, 123.8, 35.7, 34.4, 31.9, 31.3 **HRMS-ESI** (*m/z*): calculated $C_{52}H_{41}N_5O_7Re$ $[M-PF_6]^+$: 1034.2563, found 1034.2561

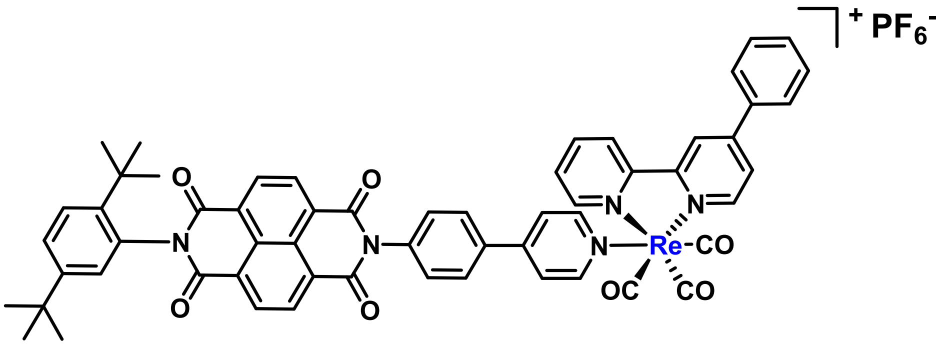

Phbpy-Re-PyPhNDI

In a nitrogen filled glove box, an oven dried pressure flask with a magnetic stirrer was filled with **Phby-Re-Cl** (0.05 g, 0.01 mmol), **DtB-NDI-PhPy** (0.06 g, 0.10 mmol), $AgPF_6$ (0.03 mg, 0.13 mmol), and dichloromethane (40 mL). The pressure flask was capped and brought out of the glovebox and heated to 80 °C overnight. The solution was cooled to room temperature and filtered through celite. The solvent was removed under reduced pressure and then column chromatographed on silica using a gradient between dichloromethane and Acetone (100 → 90:10). The compound was then dissolved in minimal dichloromethane and layered with diethyl ether. Upon filtering, a bright yellow compound was obtained (0.03 g, 40%). **^{1}H NMR** (500 MHz, Chloroform-*d*) δ 9.10 (d, *J* = 6.5 Hz, 1H), 9.06 (d, *J* = 5.8 Hz, 1H), 8.87 – 8.82 (m, 4H), 8.76 – 8.73 (d, *J* = 9.0 Hz, 1H), 8.71 (d, *J* = 1.8 Hz, 1H), 8.36 (td, *J* = 8.0, 1.6 Hz, 1H), 8.26 – 8.21 (m, 2H), 7.93 – 7.87 (m, 3H), 7.81 – 7.77 (m, 2H), 7.76 – 7.72 (m, 1H), 7.69 – 7.65 (m, 2H), 7.63 – 7.55 (m, 4H), 7.50 – 7.47 (m, 1H), 7.45 – 7.41 (m, 2H), 7.01 (d, *J* = 2.2 Hz, 1H), 1.33 (s, 9H), 1.27 (s, 9H) **^{13}C NMR** (126 MHz, $CDCl_3$) δ 163.8, 162.9, 156.3, 156.0, 153.9, 152.7, 152.0, 151.0, 150.5, 143.8, 141.8, 136.9, 136.3, 135.1, 132.0, 131.7, 131.6, 131.5, 129.9, 129.9, 129.1, 128.9, 128.5, 127.7, 127.6, 127.5, 127.4, 126.8, 126.3, 126.1, 125.0, 123.5, 35.7, 34.4, 31.8, 31.3.

HRMS-ESI (*m/z*): calculated $C_{71}H_{71}N_5O_7Re$ $[M-PF_6]^+$: 1292.4911, found 1292.4900

Transient absorption data and SVD/global fitting

Gaps in the spectra shown are due to either scattering of the pump or idler beam, or regions not covered by the detectors.

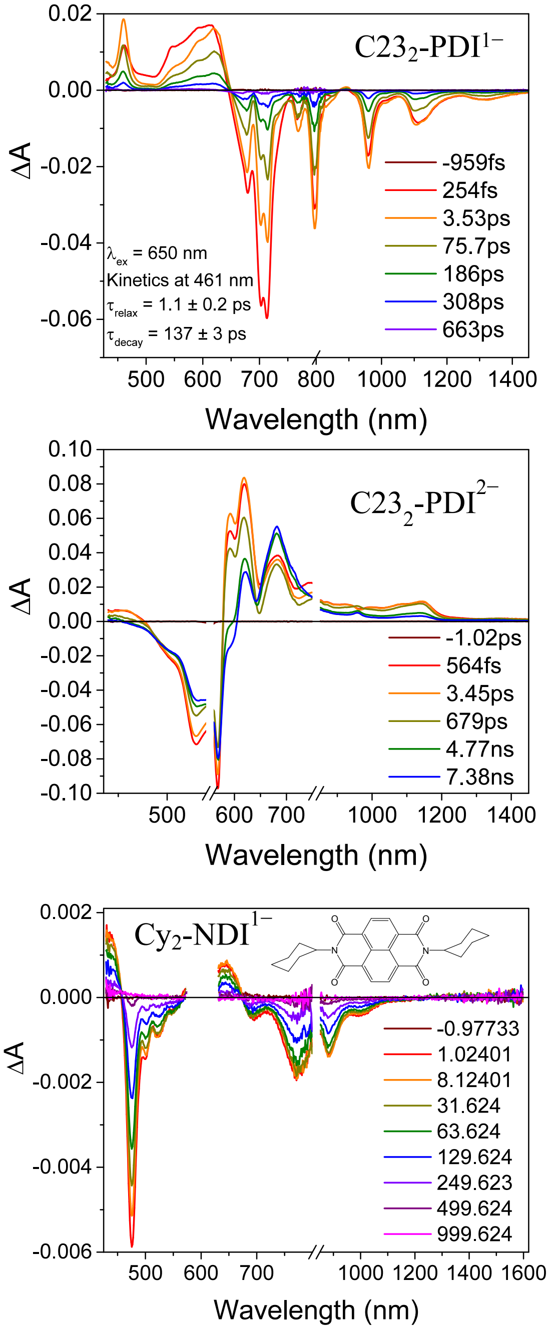

Figure S1. From top to bottom: Visible and NIR transient absorption spectra of **$C23_2$-PDI^{1-}** (λ_{ex} = 650 nm), **$C23_2$-PDI^{2-}** (λ_{ex} = 571 nm) and **Cy_2-NDI^{1-}** (λ_{ex} = 605 nm).

PDI^{1-}-Phbpy-Re-Py

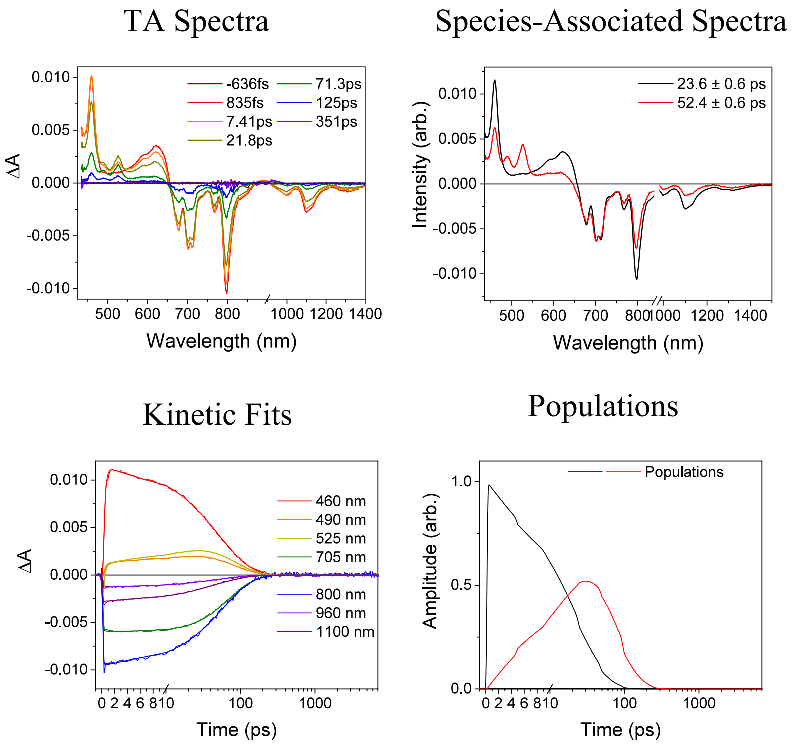

Figure S2. Transient absorption spectra, species-associated spectra, global multiple-wavelength kinetic traces and fits, and populations of each species for **PDI^{1-}-Phbpy-Re-Py** (λ_{ex} = 950 nm).

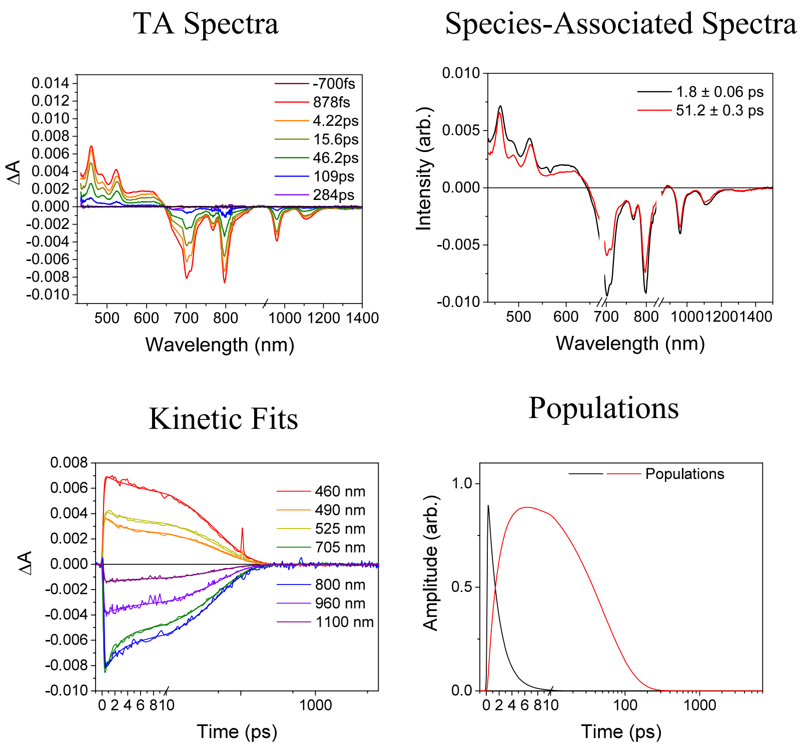

Figure S3. Transient absorption spectra, species-associated spectra, global multiple-wavelength kinetic traces and fits, and populations of each species for **PDI^{1-}-Phbpy-Re-Py** (λ_{ex} = 680 nm).

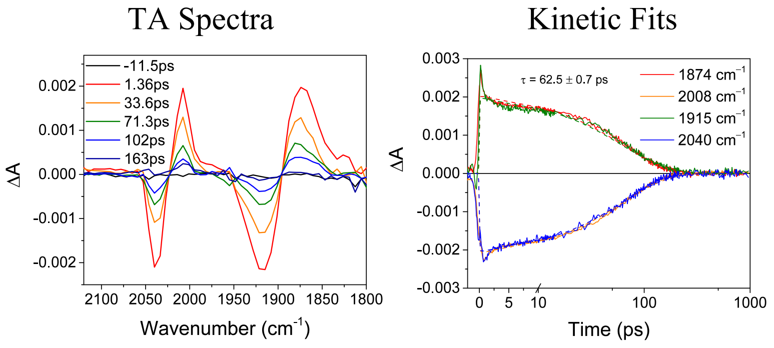

Figure S4. Time-resolved IR spectra and global multiple-wavelength kinetic traces and fits for **PDI^{1-}-Phbpy-Re-Py** (λ_{ex} = 700 nm).

PDI^{2-}-Phbpy-Re-Py

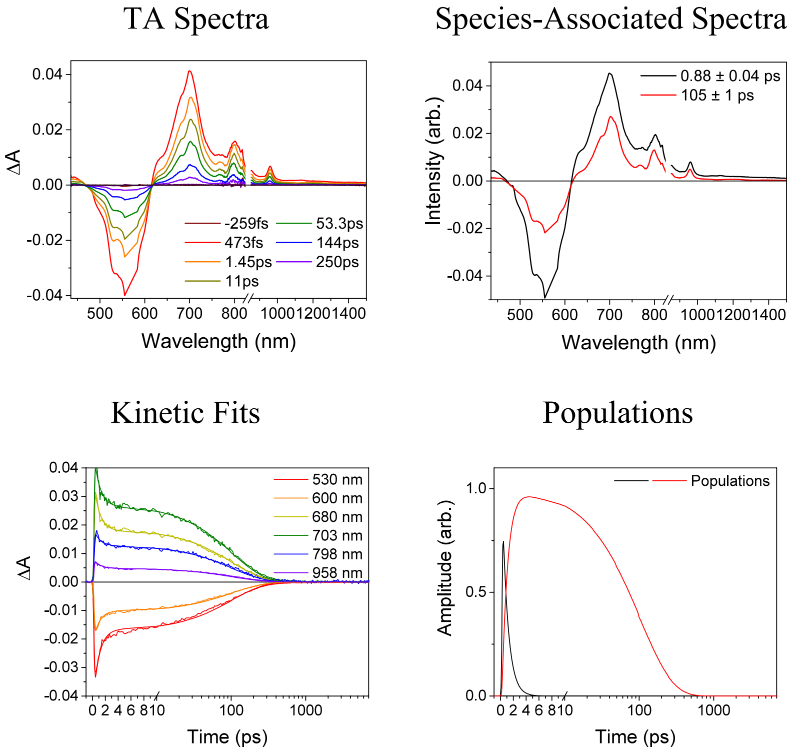

Figure S5. Transient absorption spectra, species-associated spectra, global multiple-wavelength kinetic traces and fits, and populations of each species for **PDI^{2-}-Phbpy-Re-Py** (λ_{ex} = 570 nm).

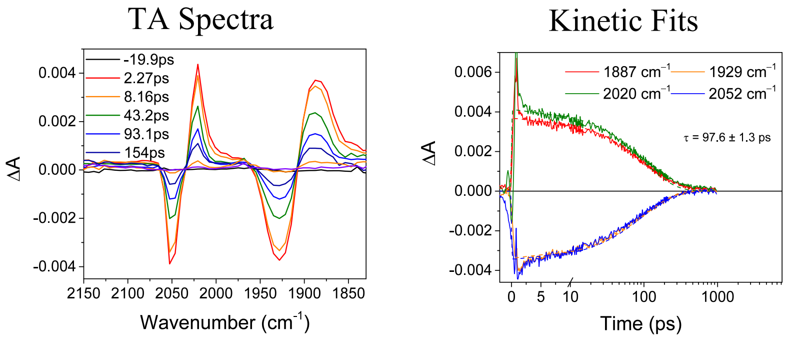

Figure S6. Time-resolved IR spectra and global multiple-wavelength kinetic traces and fits for **PDI^{2-}-Phbpy-Re-Py** (λ_{ex} = 570 nm).

NDI^{1-}-Phbpy-Re-Py

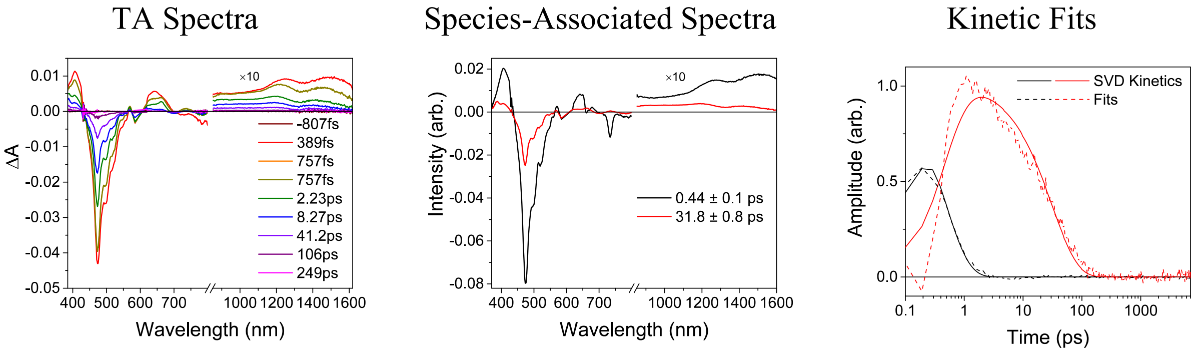

Figure S7. Transient absorption spectra, species-associated spectra, and singular value decomposition kinetic traces and fits for **NDI^{1-}-Phbpy-Re-Py** (λ_{ex} = 605 nm).

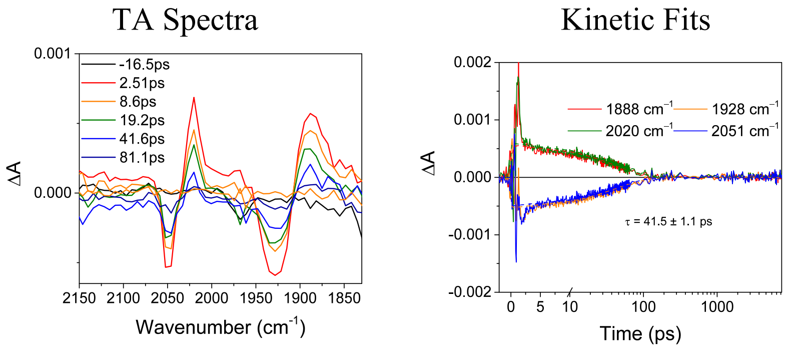

Figure S8. Time-resolved IR spectra and global multiple-wavelength kinetic traces and fits for **NDI^{1-}-Phbpy-Re-Py** (λ_{ex} = 605 nm).

Phbpy-Re-PyPhPDI^{1-}

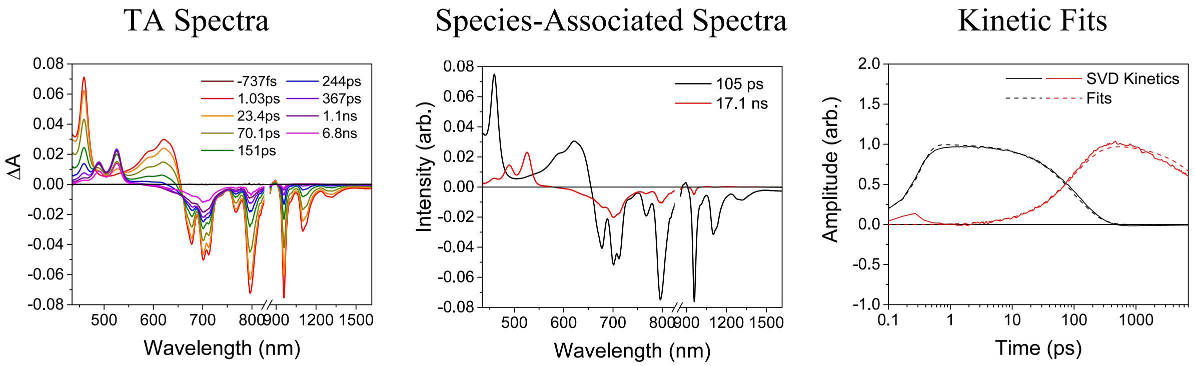

Figure S9. Transient absorption spectra, species-associated spectra, and singular value decomposition kinetic traces and fits for **Phbpy-Re-PyPhPDI^{1-}** (λ_{ex} = 950 nm).

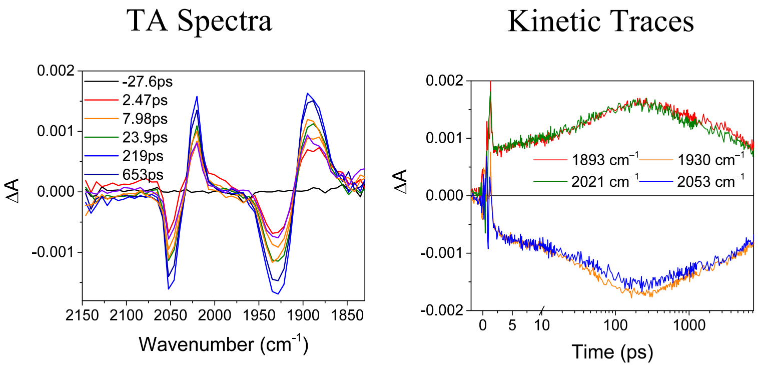

Figure S10. Time-resolved IR spectra and global multiple-wavelength kinetic traces and fits for **Phbpy-Re-PyPhPDI^{1-}** (λ_{ex} =705 nm).

Phbpy-Re-PyPhPDI^{2-}

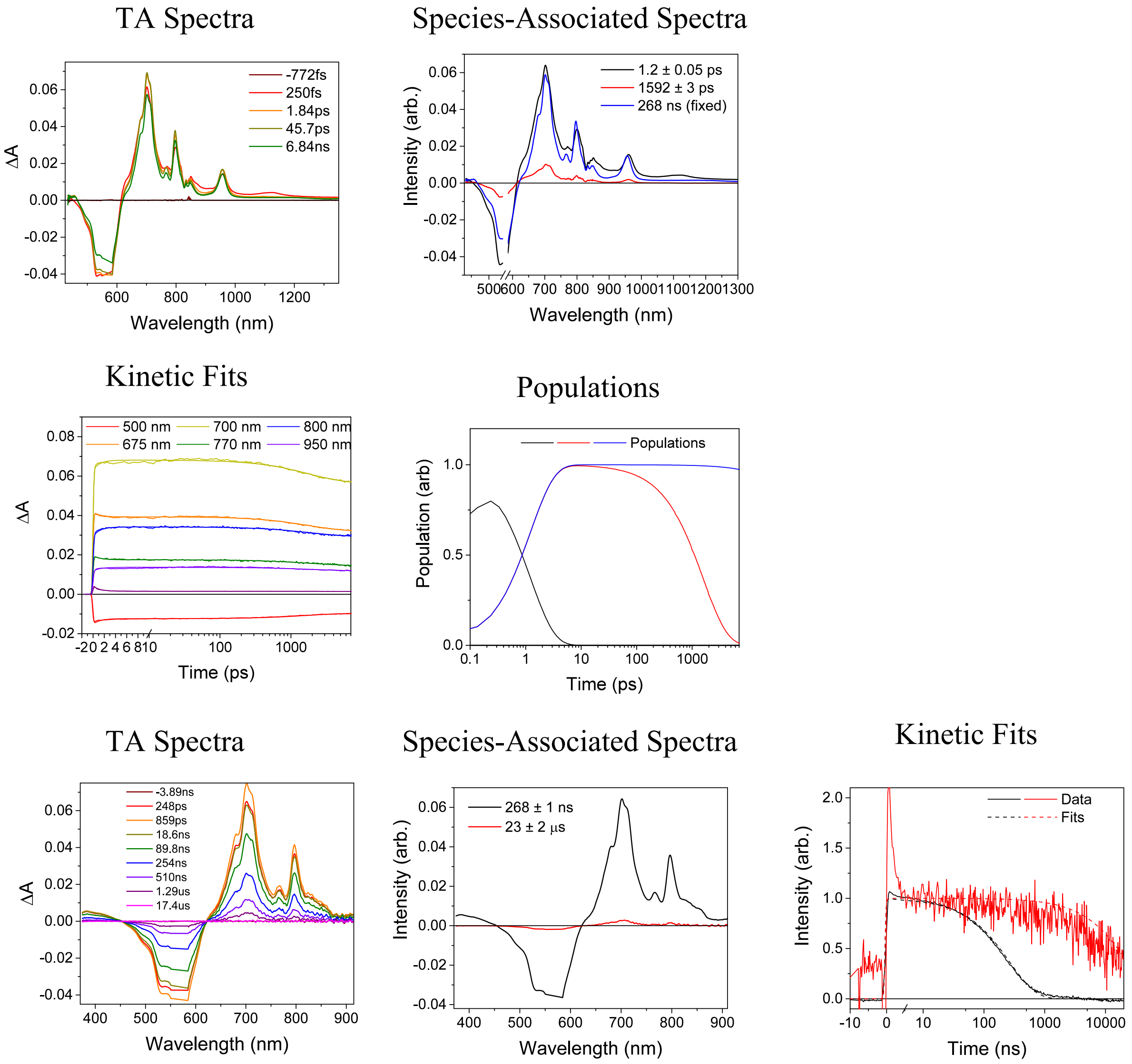

Figure S11. *Left column*: Transient absorption spectra for **Phbpy-Re-PyPhPDI^{2-}** (λ_{ex} = 570 nm). *Middle column*: Species-associated spectral fits to the data. *Right column*: Multiple-wavelength kinetic traces and fits (at short times) and singular value decomposition kinetic traces and fits (at long times) to the kinetic data based on the species-associated fits and lifetimes shown.

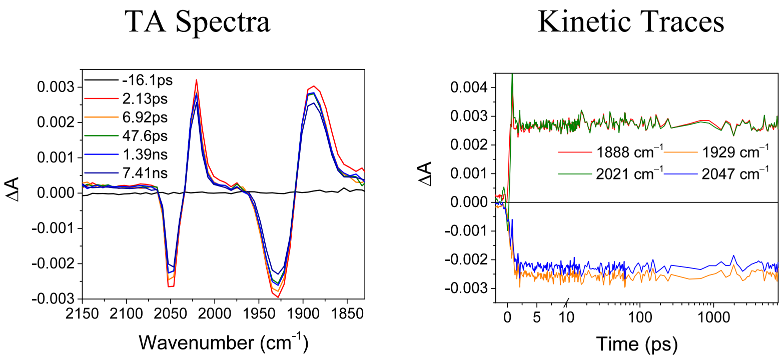

Figure S12. Time-resolved IR spectra and global multiple-wavelength kinetic traces and fits for **Phbpy-Re-PyPhPDI**$^{2-}$ (λ_{ex} = 570 nm)

Phbpy-Re-PyPhNDI^{1-}

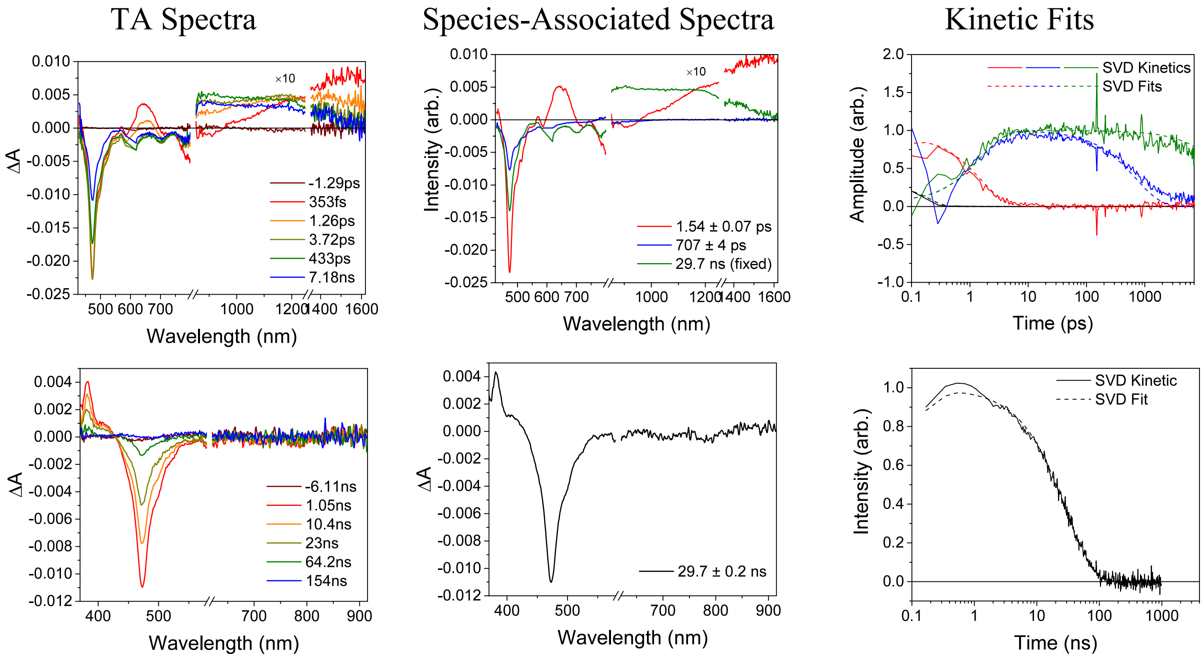

Figure S13. *Left column*: Transient absorption spectra for **Phbpy-Re-PyPhNDI^{1-}** (λ_{ex} = 605 nm). *Middle column*: Species-associated spectral fits to the data. *Right column*: Multiple-wavelength kinetic traces and fits (at short times) and singular value decomposition kinetic traces and fits (at long times) to the kinetic data based on the species-associated fits and lifetimes shown.

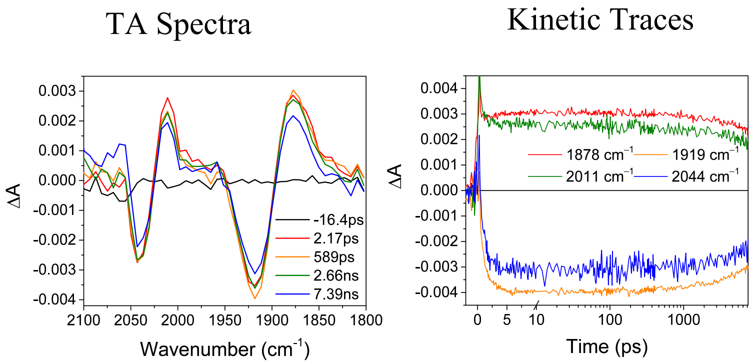

Figure S14. Time-resolved IR spectra and multiple-wavelength kinetic traces for **Phbpy-Re-PyPhNDI^{1-}** (λ_{ex} = 605 nm)

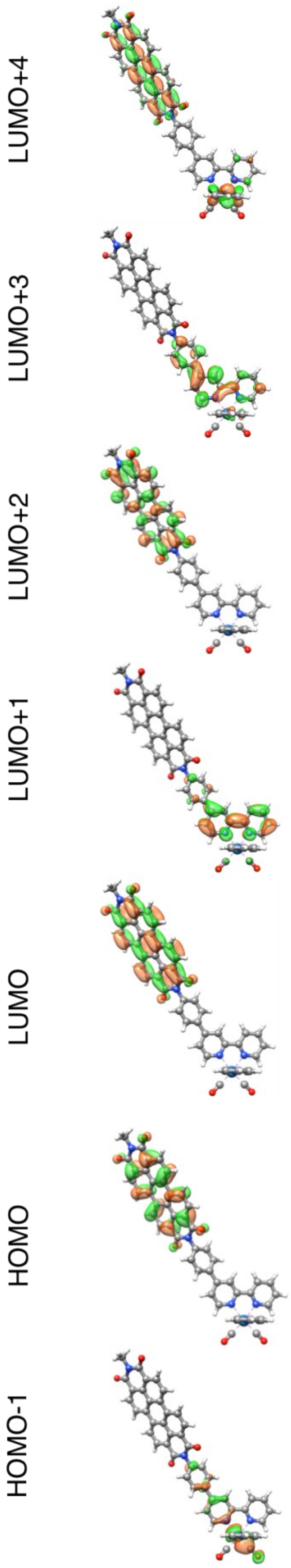

Figure S15. Frontier orbitals for the PDI−[Re(Phbpy)(py)$(CO)_3]^+$ dyad with isodensity=0.03

Table S2. Relevant orbital energies in eV for all species as non-reduced singlets.

	**$[Re(Phbpy)(py)(CO)_3]^+$**	**Dyad**	**PDI**
**LUMO+3**		−1.88	
**LUMO+2**		−1.89	−1.67
**LUMO+1**	−1.85	−2.66	−1.85
**LUMO**	−2.65	−3.42	−3.39
**HOMO**	−6.46	−5.88	−5.85
**HOMO−1**		−6.47	−7.26

Table S3. The wavelengths λ, oscillator strengths *f*, and dominant orbital characters for the 10 lowest−energy calculated vertical electronic transitions of $[Re(Phbpy)(py)(CO)_3]^+$.

**λ [nm]**	***f***	**Primary Transition Character**	**%**	**Secondary Transition Character**	**%**
394.7	0.040	HOMO→LUMO	70	HOMO−1→LUMO	28
383.4	0.117	HOMO−1→LUMO	67	HOMO→LUMO	24
369.7	0.044	HOMO−2→LUMO	91	HOMO−1→LUMO	4
317.2	0.208	HOMO−3→LUMO	76	HOMO−5→LUMO	8
308.3	0.142	HOMO→LUMO+1	56	HOMO−3→LUMO	14
304.3	0.019	HOMO−1→LUMO+1	39	HOMO−4→LUMO	21
302.2	0.016	HOMO−4→LUMO	36	HOMO−1→LUMO+1	33
301.3	0.046	HOMO→LUMO+2	39	HOMO−5→LUMO	22
295.8	0.200	HOMO−5→LUMO	35	HOMO−1→LUMO+2	26
295.5	0.089	HOMO−1→LUMO+2	33	HOMO→LUMO+2	16

Table S4. The calculated vibronic absorption maximum peak wavelengths λ of the first three electronic transitions, and the vertical wavelengths λ, oscillator strengths *f*, and dominant orbital characters for the 10 lowest−energy calculated electronic transitions of the reduced PDI^- radical.

**$\lambda_{vibronic}$ [nm]**	**λ [nm]**	***f***	**Primary Transition Character**	**%**	**Secondary Transition Character**	**%**
974.4	844.3	0.032	βHOMO→LUMO	74	αLUMO→LUMO+1	26
688.4	668.6	0.052	αLUMO→LUMO+2	97	N/A	0
717.1	621.4	0.885	αLUMO→LUMO+1	72	βHOMO→LUMO	24
	532.5	0.001	αLUMO→LUMO+3	97	N/A	0
	462.7	0.000	αLUMO→LUMO+4	39	βHOMO→LUMO+2	18
	442.2	0.000	βHOMO→LUMO+1	33	αHOMO→LUMO+1	29
	419.7	0.000	αLUMO→LUMO+4	48	βHOMO→LUMO+2	13
	400.2	0.000	βHOMO−1→LUMO	92	βHOMO−1→LUMO+1	4
	399.0	0.000	βHOMO−2→LUMO	92	βHOMO−2→LUMO+1	4
	394.3	0.000	βHOMO−3→LUMO	52	βHOMO−4→LUMO	26

Excited-state equilibrium in PDI^{1-}-Phbpy-Re-Py

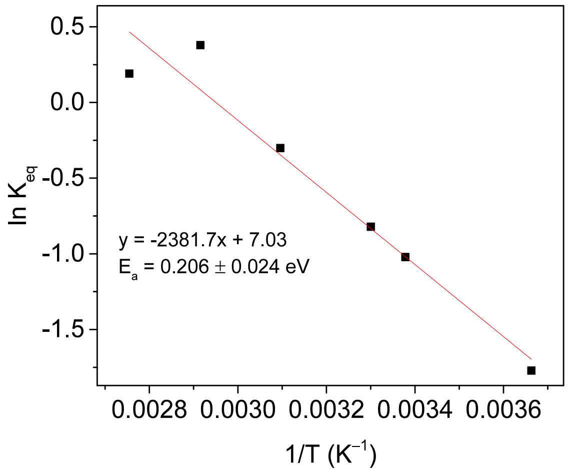

Figure S16. Plot of ln K_{eq} vs. 1/T derived from the $[PDI^{0}]:[PDI^{1-*}]$ ratio in the SVD spectra of variable-temperature TA experiments on **PDI^{1-}-Phbpy-Re-Py**.

Calculated electron density of reduced Re(Phbpy$^{\bullet-}$)$(CO)_3$(PyPh)

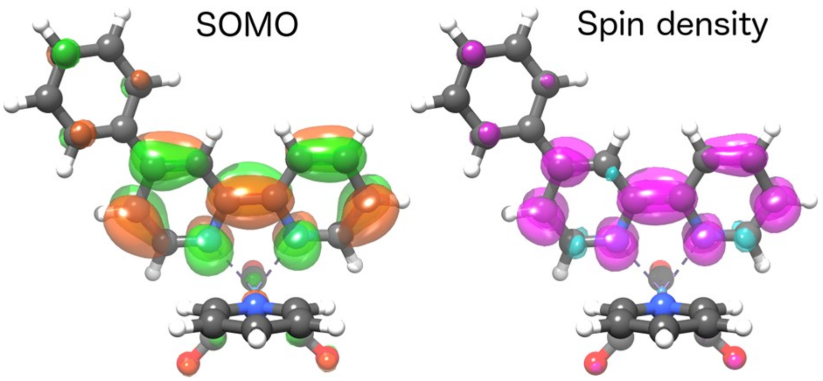

Figure S17. Calculated SOMO (isovalue=0.03) and spin density (isovalue=0.002, magenta for spin up and teal for spin down) of the Re(Phbpy$^{\bullet-}$)$(CO)_3$(PyPh) doublet anion, calculated with B3LYP/Def2TZVP//Def2SVP showing that the reducing electron density is largest on the bpy, with some density extending onto the 4-phenyl group.

Complete Reference 25 from the Main Text:

Shao, Y.; Gan, Z.; Epifanovsky, E.; Gilbert, A. T. B.; Wormit, M.; Kussmann, J.; Lange, A. W.; Behn, A.; Deng, J.; Feng, X.; Ghosh, D.; Goldey, M.; Horn, P. R.; Jacobson, L. D.; Kaliman, I.; Khaliullin, R. Z.; Kuś, T.; Landau, A.; Liu, J.; Proynov, E. I.; Rhee, Y. M.; Richard, R. M.; Rohrdanz, M. A.; Steele, R. P.; Sundstrom, E. J.; Woodcock, H. L.; Zimmerman, P. M.; Zuev, D.; Albrecht, B.; Alguire, E.; Austin, B.; Beran, G. J. O.; Bernard, Y. A.; Berquist, E.; Brandhorst, K.; Bravaya, K. B.; Brown, S. T.; Casanova, D.; Chang, C.-M.; Chen, Y.; Chien, S. H.; Closser, K. D.; Crittenden, D. L.; Diedenhofen, M.; DiStasio, R. A.; Do, H.; Dutoi, A. D.; Edgar, R. G.; Fatehi, S.; Fusti-Molnar, L.; Ghysels, A.; Golubeva-Zadorozhnaya, A.; Gomes, J.; Hanson-Heine, M. W. D.; Harbach, P. H. P.; Hauser, A. W.; Hohenstein, E. G.; Holden, Z. C.; Jagau, T.-C.; Ji, H.; Kaduk, B.; Khistyaev, K.; Kim, J.; Kim, J.; King, R. A.; Klunzinger, P.; Kosenkov, D.; Kowalczyk, T.; Krauter, C. M.; Lao, K. U.; Laurent, A. D.; Lawler, K. V.; Levchenko, S. V.; Lin, C. Y.; Liu, F.; Livshits, E.; Lochan, R. C.; Luenser, A.; Manohar, P.; Manzer, S. F.; Mao, S.-P.; Mardirossian, N.; Marenich, A. V.; Maurer, S. A.; Mayhall, N. J.; Neuscamman, E.; Oana, C. M.; Olivares-Amaya, R.; O'Neill, D. P.; Parkhill, J. A.; Perrine, T. M.; Peverati, R.; Prociuk, A.; Rehn, D. R.; Rosta, E.; Russ, N. J.; Sharada, S. M.; Sharma, S.; Small, D. W.; Sodt, A. *Mol. Phys.* **2015**, *113*, 184.

References

1. N. G. Connelly and W. E. Geiger, *Chemical Reviews*, 1996, **96**, 877-910.
2. D. Gosztola, M. P. Niemczyk, W. Svec, A. S. Lukas and M. R. Wasielewski, *The Journal of Physical Chemistry A*, 2000, **104**, 6545-6551.
3. A. D. Becke, *Journal of chemical physics*, 1993, **98**, 5648-5652.
4. M. J. T. Gaussian 09 Revision D.01; Frisch, G. W.; Schlegel, H. B.; Scuseria, G. E.; Robb, M. A.; Cheeseman, J. R.; Scalmani, G.; Barone, V.; Mennucci, B.; Petersson, G. A.; Nakatsuji, H.; Caricato, M.; Li, X.; Hratchian, H. P.; Izmaylov, A. F.; Bloino, J.; Zheng, G.; Sonnenberg, J. L.; Hada, M.; Ehara, M.; Toyota, K.; Fukuda, R.; Hasegawa, J.; Ishida, M.; Nakajima, T.; Honda, Y.; Kitao, O.; Nakai, H.; Vreven, T.; Montgomery, J. A., Jr.; Peralta, J. E.; Ogliaro, F.; Bearpark, M.; Heyd, J. J.; Brothers, E.; Kudin, K. N.; Staroverov, V. N.; Kobayashi, R.; Normand, J.; Raghavachari, K.; Rendell, A.; Burant, J. C.; Iyengar, S. S.; Tomasi, J.; Cossi, M.; Rega, N.; Millam, N. J.; Klene, M.; Knox, J. E.; Cross, J. B.; Bakken, V.; Adamo, C.; Jaramillo, J.; Gomperts, R.; Stratmann, R. E.; Yazyev, O.; Austin, A. J.; Cammi, R.; Pomelli, C.; Ochterski, J. W.; Martin, R. L.; Morokuma, K.; Zakrzewski, V. G.; Voth, G. A.; Salvador, P.; Dannenberg, J. J.; Dapprich, S.; Daniels, A. D.; Farkas, Ö.; Foresman, J. B.; Ortiz, J. V.; Cioslowski, J.; Fox, D. J. Gaussian, Inc., Wallingford CT, 2009.
5. F. Weigend and R. Ahlrichs, *Physical Chemistry Chemical Physics*, 2005, **7**, 3297-3305.
6. A. V. Marenich, C. J. Cramer and D. G. Truhlar, *Journal of Physical Chemistry B*, 2009, **113**, 6378-6396.
7. R. Improta, V. Barone, G. Scalmani and M. J. Frisch, *Journal of chemical physics*, 2006, **125**, 054103.
8. R. Improta, G. Scalmani, M. J. Frisch and V. Barone, *Journal of chemical physics*, 2007, **127**, 074504.
9. G. Scalmani and M. J. Frisch, *Journal of chemical physics*, 2010, **132**, 114110.
10. R. Bauernschmitt and R. Ahlrichs, *Chemical Physics Letters*, 1996, **256**, 454-464.
11. M. E. Casida, C. Jamorski, K. C. Casida and D. R. Salahub, *Journal of chemical physics*, 1998, **108**, 4439-4449.
12. F. Furche and R. Ahlrichs, *Journal of chemical physics*, 2002, **117**, 7433-7447.
13. G. Scalmani, M. J. Frisch, B. Mennucci, J. Tomasi, R. Cammi and V. Barone, *Journal of chemical physics*, 2006, **124**, 094107.
14. R. E. Stratmann, G. E. Scuseria and M. J. Frisch, *Journal of Chemical Physics*, 1998, **109**, 8218-8224.
15. C. Van Caillie and R. D. Amos, *Chemical physics letters*, 1999, **308**, 249-255.
16. C. Van Caillie and R. D. Amos, *Chemical Physics Letters*, 2000, **317**, 159-164.
17. V. Barone, J. Bloino and M. Biczysko, *September*, 2009, **2**, 2009.
18. I. The Mathworks, *Journal*.
19. E. R. Henry and J. Hofrichter, in *Methods in Enzymology*, Academic Press, 1992, vol. Volume 210, pp. 129-192.
20. M. N. Berberan-Santos and J. M. G. Martinho, *Journal of Chemical Education*, 1990, **67**, 375.
21. M. J. Robb, B. Newton, B. P. Fors and C. J. Hawker, *The Journal of Organic Chemistry*, 2014, **79**, 6360-6365.

22. M. J. Ahrens, R. F. Kelley, Z. E. X. Dance and M. R. Wasielewski, *Physical Chemistry Chemical Physics*, 2007, **9**, 1469-1478.
23. D. A. M. Egbe, A. M. Amer and E. Klemm, *Designed Monomers and Polymers*, 2001, **4**, 169-175.
24. M. Zalas, B. Gierczyk, M. Cegłowski and G. Schroeder, *Chemical Papers*, 2015, **66**, 733-740.
